# Risk factors for cutaneous immune-related adverse events: a systematic scoping review

**DOI:** 10.3389/fimmu.2026.1722781

**Published:** 2026-04-16

**Authors:** Zhenzhen Su, Liyan Zhang, Xuemin Lian, Yixuan Wang

**Affiliations:** 1 Key Laboratory of Carcinogenesis and Translational Research (Ministry of Education), Nursing Department, Peking University Cancer Hospital & Institute, Beijing, China; 2 Key Laboratory of Carcinogenesis and Translational Research (Ministry of Education), Department of Gastrointestinal Oncology, Peking University Cancer Hospital & Institute, Beijing, China; 3 Department of Health and Medical, Tianjin Medical University General Hospital, Tianjin, China; 4 School of Nursing, Peking University, Beijing, China

**Keywords:** cancer, cutaneous, immune-related adverse events, immunotherapy, review, risk factors

## Abstract

**Objective:**

To comprehensively summarize risk factors and explore potential mechanisms associated with cutaneous immune-related adverse events (cirAEs) in cancer patients treated with immune checkpoint inhibitors (ICIs).

**Method:**

Conducted in accordance with the PRISMA-ScR guidelines, this review included studies published in English and Chinese that investigated cirAEs risk factors in cancer patients receiving ICIs. A scoping review with systematic search criteria was conducted using PubMed, Embase, Cochrane, Web of Science, ProQuest, CINAHL, CNKI, Wanfang Data, VIP, and SinoMed from database inception to December to December 31, 2023.

**Results:**

4905 studies were identified and 50 studies were included in this review, encompassing 198,514 participants. Among these, 12.5% experienced at least one cirAEs, with maculopapular rash, pruritus, and unspecified rash being the most common subtypes. A total of 68 distinct risk factors were identified from these studies, categorized into three primary risk domains: demographic factors, clinical characteristics, and biomarkers, including age, gender, BMI, smoking history, specific treatment regimens (e.g., camrelizumab), tumor type (e.g., melanoma), eosinophil count, and cytokine levels, among others. The odds ratios (OR) for reported risk factors demonstrated significant associations with specific cirAEs subtypes, with melanoma patients exhibiting higher risk for multiple cirAEs subtypes. However, there was significant variability in the quality of reporting for these risk factors, emphasizing the need for improved consistency and accuracy in data reporting.

**Conclusions:**

A variety of demographic, clinical, and biomarker-related factors contribute to the development of cirAEs. Characterizing these risk factors can address clinical needs for cirAE identification, while further mechanistic studies are needed to enhance management strategies. However, there is a limited amount of high-quality prospective evidence on these risk factors, and the quality of reporting on immunotherapy-related adverse events is inconsistent. Future research should focus on validating clinically valuable risk factors and interrogating mechanisms underlying cirAEs emergence.

## 1 Introduction

The emergence of immune checkpoint inhibitors has ushered in a new era for modern cancer treatment (1). It has been shown that ICIs, such as programmed cell death-1/ligand 1 (PD-1/PD-L1) and cytotoxic T lymphocyte antigen-4 (CTLA-4) inhibitors, prolong the survival of patients with various solid tumors and hematological malignancies (2). However, due to the lack of specificity, disinhibition of T/B-cell function by ICIs can lead to immune activation in other systems, resulting in immune-related adverse events (irAEs) (3). Cutaneous immune-related adverse events (cirAEs) are the most common and earliest irae, occurring in patients as early as day 1 after treatment initiation and as late as 1 to 2 years after treatment, and are related to the choice of treatment regimen (4–7).

Various morphologic patterns of cirAEs have emerged, including maculopapular, psoriasis, pruritus, vitiligo, and reactive cutaneous capillary endothelial proliferation (RCCEP), with incidence rates ranging from 30% to over 50% (8). Although most cirAEs are mild and can be managed with local therapy, serious or life-threatening reactions can occur, such as bullous pemphigoid (BP) and Stevens-Johnson syndrome/toxic epidermal necrolysis (SJS/TEN) (9, 10). These cirAEs may disrupt cancer therapy, necessitate hospitalization, or even cause death (11). To reduce the disruptions caused by these severe events, there is an ongoing need to better understand contributing risk factors.

Recently, advances in biostatistical methods and informatics approaches have suggested factors that may correlate with risk of immunotoxicities, including cirAEs, in several cancer settings (12–15). Factors, including demographic characteristics such as older age, clinical features such as camrelizumab therapy, and biomarkers such as low NLR at baseline, have all been reported to increase the risk of cirAEs (12, 14, 16, 17). However, the reported risk factors are inconsistent, and some factors may be reported in only one study. To strengthen understanding of contributors to cirAEs risk, a comprehensive collation of existing research is needed.

Therefore, the overall aim of our scoping review is to summarize risk factors and discuss possible mechanisms to improve patient safety.

## 2 Methods

This scoping review was conducted in accordance with the PRISMA-ScR (Preferred Reporting Items for Systematic Reviews and Meta-Analyses extension for Scoping Reviews) checklist framework (18, 19). The PRISMA-ScR checklist is provided in **Supplementary Material (**Appendix 1).

### 2.1 Eligibility criteria

An extensive search and broad inclusion criteria were used to capture all relevant studies, limiting to: 1) cancer patients who had received at least one ICI treatment, 2) included studies mentioned risk factors associated with the incidence of any cirAEs, 3) both experimental and observational designs were included, such as randomized controlled trials (RCTs), quasi-RCTs, non-RCTs, before-after studies, interrupted time series studies, case-control studies, retrospective or prospective cohort studies, longitudinal observational studies, and Meta-analysis. Only studies published in English and Chinese were included. The details of eligibility table for inclusion and exclusion criteria were shown in **Table 1**.

**Table 1:** Inclusion and exclusion criteria.

PICOS	Inclusion criteria	Exclusion criteria
Population (P)	Cancer patients have received at least one dose ICIs treatments	Not cancer patients or not treated with ICIs therapy
Concept (C)	The included studies should mention risk factors associated with the incidence of any cirAEs.	Not cirAEs reported Risk factors only between different grades cirAEs but not non-cirAEs Risk factors of prognostic outcomes Risk factors not analyzed statistically
Context (C)	Any setting, including the patient’s home or health care facility. English/Chinese Time published from the date of database establishment to Dec 31^st^, 2023	Not in English/Chinese Time out of the inclusion criterion Duplicate report No full text
Study design (S)	Experimental design, including randomized control trial (RCT), quasi-RCT, non-RCT, before-after study and interrupted time series studies Observational design, including case control study, retrospective or prospective cohort study and longitudinal observational study Meta-analysis	Other Review, conference abstract, comment, protocol only, case report, qualitative research

### 2.2 Search strategy

We searched PubMed, Embase, Cochrane, Web of Science, ProQuest, CINAHL, CNKI, Wanfang Data, VIP, and SinoMed to identify potentially relevant studies using the following keywords: “cancer/neoplasms/tumor,” “immunotherapy/immune checkpoint inhibitors,” “adverse events/drug-related side effects and adverse reactions,” “immune-related adverse events,” and “exanthema/maculopapular/cutaneous toxicity.” Additionally, we reviewed reference lists to identify further studies of interest. The search was initiated on December 31, 2023. Details of the search terms are provided in **Supplementary Material (**Appendix 2). Endnote X9 was used to manage the evidence selection process. After screening and removing duplicates, two researchers (ZS and XL) independently reviewed the titles and abstracts, followed by full-text assessments of the remaining studies to determine eligibility. According to our eligibility criteria, articles that did not report cirAEs, or only reported risk factors between different grades of cirAEs, or did not perform statistical analysis, or excluded meaningless risk factors were excluded. Any discrepancies were resolved through discussion or consultation with a third researcher (LZ). To quantify the level of agreement between the two primary reviewers, we calculated the kappa statistic for interrater reliability. The kappa values were 0.82 for the initial screening of titles and abstracts and 0.85 for the full-text assessment, indicating a high level of agreement.

### 2.3 Data extraction

Three independent researchers (ZS, XL and YW) performed data extraction with cross-checking to ensure accuracy. Discrepancies were resolved through consensus discussions or consultation with another investigator (LZ). The data extraction included the first author, year of publication, country, study design, participants, sample size, age, follow-up duration, subtypes of cirAEs, time of occurrence, grade of cirAEs, assessment tools, results, and risk factors. All extracted data were obtained from full-text studies.

### 2.4 Results synthesis

Based on the extracted data, risk factors with similar sources and concepts were classified and discussed by two researchers, ZS and XL. For each risk factor, we calculated the percentage of included studies that measured this specific risk factor. Then, we constructed a correlation diagram between various risk factors and cirAEs to facilitate a better understanding and identification of the relationships. Finally, we additionally extracted the odds ratio (OR) for each risk factor.

## 3 Results

### 3.1 Characteristics of included studies

**Figure 1:**
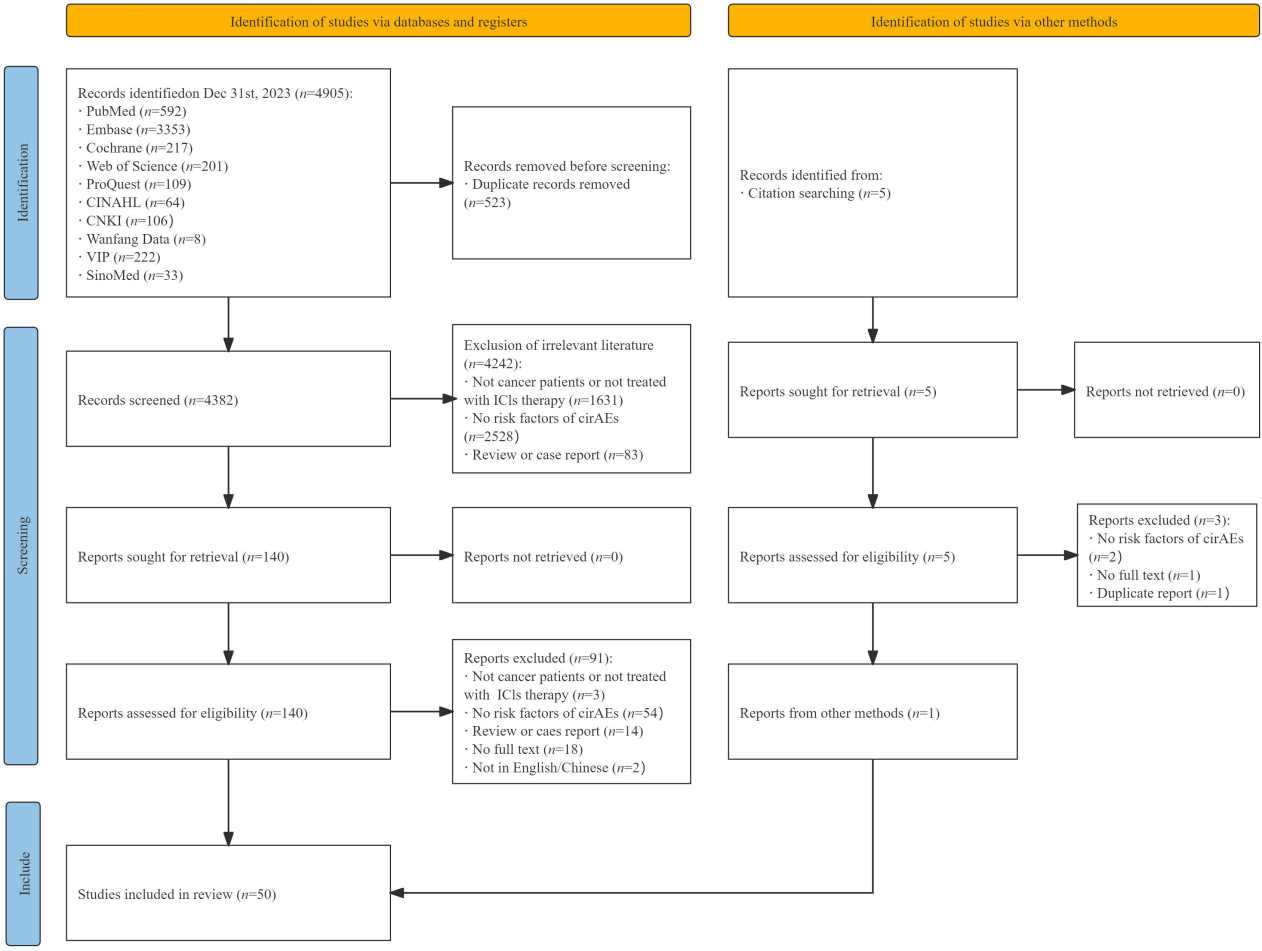
Preferred Reporting Items for Systematic Reviews and Meta-Analyses extension for Scoping Reviews (PRISMA-ScR) flowchart of the study selection process. ICIs, immune checkpoint inhibitors. CirAEs, cutaneous immune-related adverse events.

A total of 4905 studies were identified, of which 140 were retrieved for full-text review. Among these 140 studies, 2 was not reported in English or Chinese, 18 were not available in full text, 3 did not involve cancer patients or ICIs therapies, 54 did not investigate risk factors for cirAEs, 14 were reviews or case reports. Additionally, to ensure a more comprehensive search, we screened the references of the 5 reviews, and 1 met our eligibility criteria. Ultimately, 50 studies were included in this review (12–16, 20–64) (**Figure 1**).

In total, we included 50 studies involving a total of 198,514 participants in this review (range, 40-123,104). The most common study design was the case control study (48.0%) and cohort study (44.0%). In terms of cancer type, pan-cancer, melanoma, and non-small cell lung cancer were the most commonly studied types (38.0%, 30.0%, 30.0%). The most commonly used ICI treatment regimen is PD-1/L1 inhibitor therapy (54.0%). Additionally, compared with other countries, research from the USA and China was more extensive (32.0% respectively) (Appendix 3).

### 3.2 Characteristics of included cutaneous immune-related adverse events

Among all 198,514 patients, 24,814 patients (12.5%) experienced at least one cirAEs. A total of 29 studies (58.0%) reported subtypes of cirAEs, with the most common subtypes including maculopapular rash (4.1% of all patients), pruritus (3.5% of all patients), and unspecified rash (0.7% of all patients).Thirty research reports examined the severity grades of cirAEs in patients, with the majority of patients (51.0% of cirAEs patients) experiencing grades 1~2, and 2.6% of cirAEs patients reporting grades 3~4. The overall onset time of cirAEs ranged from 2 to 1,065 days after initiation of ICI therapy, with a median time of 23 to 212 days (**Table 2**).

**Table 2:** Characteristics of included cutaneous immune-related adverse events.

Terms	n	Percentage of all patients (%)	Percentage of cirAEs (%)
CirAEs	24,814	12.5	–
Subtypes			
Maculopapular	8,201	4.1	33.0
Pruritus	6,941	3.5	28.0
Unspecified rash	1,299	0.7	5.2
Vitiligo	640	0.3	2.6
Psoriasis	551	0.3	2.2
Skin eruption	390	0.2	1.6
STS/JEN	378	0.2	1.5
Eczema	236	0.1	1.0
Lichenoid	228	0.1	0.9
BP	113	0.1	0.5
Mucositis	132	0.1	0.5
RCCEP	104	0.1	0.4
Others^a^	314	0.2	1.3
Grade			
1~2	12,659	6.4	51.0
3~4	634	0.3	2.6
NR	11,521	5.8	46.4
Occurrence time (days)	Range	Median	IQR
	2~1065	23~212	[2~42, 84~1015]

CirAEs, cutaneous immune-related adverse events. SJS/TEN, Stevens-Johnson syndrome/toxic epidermal necrolysis. BP, bullous pemphigoid. RCCEP, reactive cutaneous capillary endothelial proliferation. NR, not reported. IQR, Interquartile range. ^a^Others types including granulomatous, erythema multiforme, DRESS, morbilliform, urticarial, acneiform eruption, mucositis, panniculitis, sweet syndrome, severe cutaneous adverse reaction, alopecia, xerosis, arcoidosis, Grove’s disease.

### 3.3 Classification of risk factors

Based on the existing evidence from 50 included studies, 68 similar or different risk factors were extracted (all *p* < 0.05). These risk factors were divided into 12 different domains according to their relevant characteristics. We further divided these domains into three main risk areas: demographic factors, clinical features, and biomarkers. Different risk factors were shown to be associated with different cirAEs subtypes (**Table 3**).

**Table 3:** Domains of significant risk factors of cutaneous immune-related adverse events.

Areas	Domains	Subdomains	Risk factors	CirAEs subtypes
Demographic factors				
	Basic information	Age	≥ 65	Maculopapular (29)
				- (50)
			≥ 70	BP (15)
				- (22, 47)
			> 75	- (41)
			Higher age	- (40)
		Gender	Female (post-menopausal)(age ≥52)	- (26)
			Female	- (26)
				Maculopapular (51)
			Male	- (30, 31)
		Race	White	- (53)
			Asian ancestry	- (44)
	Body status	ECOG PS	0-1	- (40, 43, 63)
		BMI	>25	- (23)
			25 < BMI ≤ 29.9 (Overweight)	- (28)
			> 30 (Obese)	- (28)
			High BMI	- (44)
	Smoking status		Smoking history	Maculopapular (59)
				- (27, 42)
			Smoking index > 400	Maculopapular (59)
Clinical features	Previous treatment history	Skin disease history	Atopic dermatitis	- (55)
			Cutaneous inflammatory skin diseases	- (24)
			Morphea	- (55)
			Psoriasis	- (55)
			Pemphigus	- (55)
		Other chronic disease history	Inflammatory/auto-immune disease	- (12)
			Atopy	- (12)
			Chronic kidney disease stage 3-4	- (41)
		Drug allergy history	Drug allergy ≥ 1	Maculopapular (51)
				Pruritus (37)
				Nonspecific rash (37)
			Drug allergies ≥ 3	- (37)
			Penicillin allergy	- (37)
		History of treatment	Chemotherapy history	Maculopapular (51)
	Treatment regimens	Therapy options	Camrelizumab	RCCEP (64)
			Anti-PD-1	- (56)
			Anti-CTLA-4 therapy	Maculopapular (34)
				Pruritus (34)
			Anti-PD-(L)1 + anti-CTLA-4	Maculopapular (34)
				Pruritus (34)
				- (56)
			ICIs + TVEC therapy	- (39)
			ICIs + chemotherapy	Pruritus (46)
			ICIs + targeted therapy	- (27)
		Treatment cycles or dose	≥2 cycles	- (27)
			Longer cycles	- (12)
			High dose of ipilimumab (10mg/kg>3mg/kg)	- (25)
		Combination drug therapy	No corticosteroid used	- (58)
			Not combined with antiangiogenic drugs	- (61)
			Not use aspirin	Maculopapular (60)
				SJS (60)
				Vitiligo (60)
	Cancer types	Nonskin cancer	Renal cell carcinoma	- (56)
		Skin cancer	Melanoma	BP (15)
				Maculopapular (46)
				Vitiligo (46)
				Multiple toxicities (46)
				- (56)
			Non-acral cutaneous melanoma	- (45)
			Nonmelanoma skin cancer	BP (15)
	TNM stage		≥ 2	Maculopapular (51)
Biomarkers	Blood cell counts and ratios		Higher eosinophil count (> 0.5 g/L)	- (12)
			AEC > 0.205 × 10^9/L	- (21)
			Eosinophil (EOS%) >1.75%	- (36)
			Higher ALC	- (57)
			Lower NLR	- (40, 43, 63)
			NLR <2.86	- (32)
			PLR < 156	- (38)
			Lower LDH	- (40)
			CD4+/CD8+ ratio < 1.10 at baseline	- (58)
			CD4 + PLT + high percentage of circulating leukocyte-PLT complexes	- (62)
	Cytokines		Higher BTLA	Maculopapular (16)
			Higher GM-CSF	Maculopapular (16)
			Higher IL-4	Maculopapular (16)
			CCL19 increased from baseline to day 42 (threshold of 132–9 pg/mL)	Vitiligo (33)
			High Ang-1	- (54)
			High CD40L	- (54)
	Immunomodulatory molecules		Higher TIM-3	Maculopapular (58)
			Higher PD-(L)1 expression	Maculopapular (58)
			PD-L1 expression ≥ 1%	- (16)
	Autoantibodies		Higher anti-BP 180 IgG	BP (35)
	Genetic markers		HLADRB1*11:01	Pruritus (13)

CirAEs, cutaneous immune-related adverse events. BP, bullous pemphigoid. ECOG PS, Eastern Cooperative Oncology Group performance status. BMI, body mass index. RCCEP, reactive cutaneous capillary endothelial proliferation. PD-(L)-1, programmed cell death (ligand)-1. CTLA-4, cytotoxic T lymphocyte antigen-4. TVEC, talimogene laherparepvec. SJS, Stevens-Johnson syndrome. TNM, tumor node metastasis. AEC, absolute eosinophil count. ALC, absolute lymphocyte count. NLR, neutrophil to lymphocyte ratio. PLR, platelet-to-lymphocyte ratio. LDH, lactate dehydrogenase. PLT, platelet. BTLA, B- and T-lymphocyte attenuator. GM-CSF, granulocyte-macrophage colony stimulating factor. IL-4, interleukin-4. Ang-1, angiopoietins-1. TIM-3, T cell immunoglobulin and mucin domain 3. -, unspecified cirAEs subtypes, overall cirAEs.

Demographic factors, including age, sex, BMI, smoking status, and ECOG performance status, have been shown in studies to be associated with overall cirAEs, maculopapular rash, BP, and vitiligo. Clinical features, encompassing previous treatment history (e.g., skin disease, allergies, chemotherapy), treatment regimens (e.g., anti-CTLA-4 therapy, combination immunotherapy, camrelizumab), tumor type (e.g., melanoma), and TNM stage, were associated with overall cirAEs, maculopapular rash, pruritus, BP, vitiligo, reactive cutaneous capillary endothelial proliferation (RCCEP), and Stevens-Johnson syndrome (SJS). Biomarkers, including blood cell counts and ratios (e.g., eosinophil count, NLR, PLR), cytokines (e.g., BTLA, GM-CSF, IL-4), immunomodulatory molecules (e.g., TIM-3, PD-L1), autoantibodies (anti-BP180 IgG), and genetic markers (HLA-DRB1*11:01), were associated with overall cirAEs, maculopapular rash, BP, pruritus, and vitiligo (**Table 3**).

### 3.4 Odds ratio of risk factors

**Figure 2:**
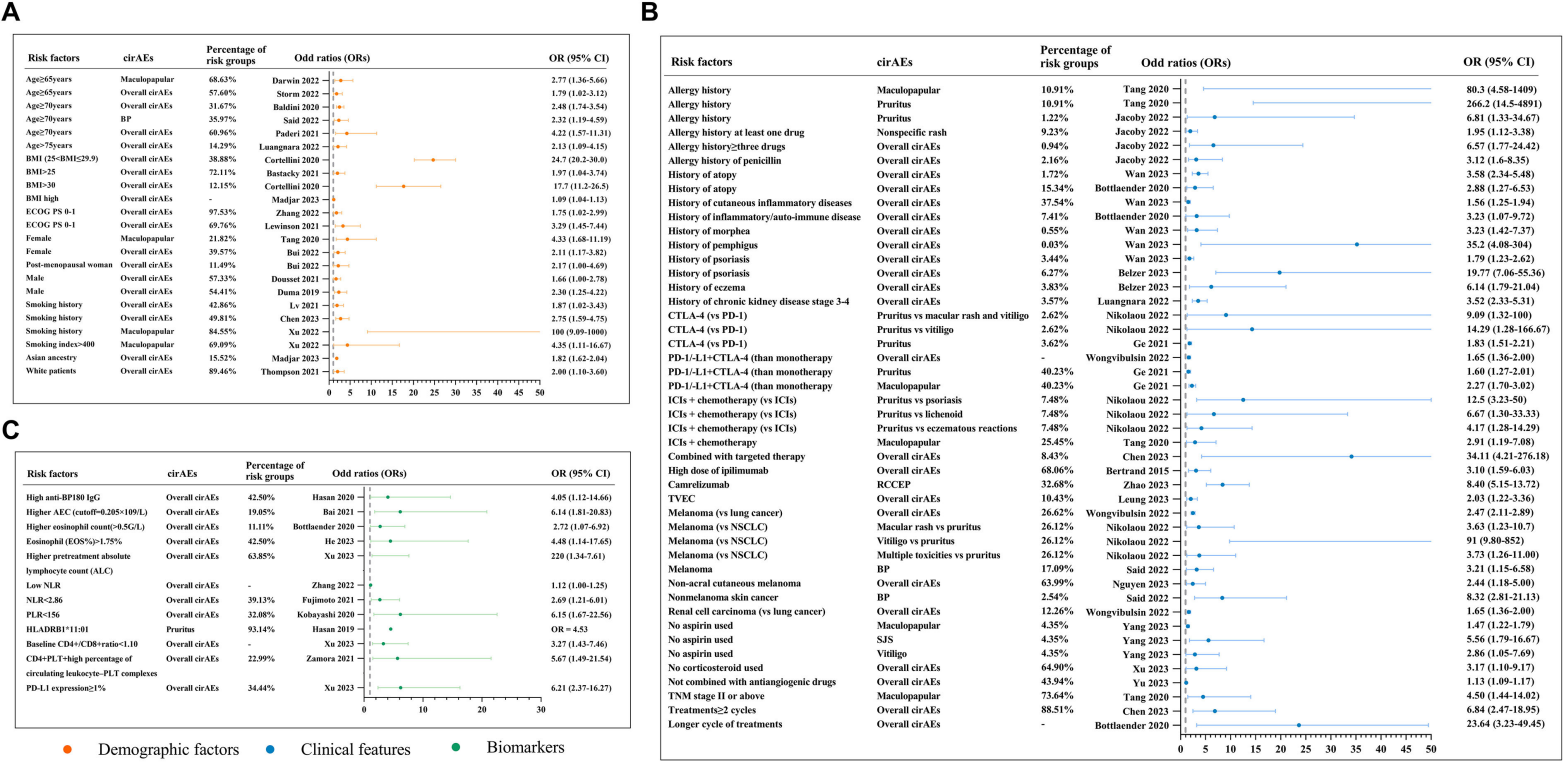
**| (A)** Odds ratios and percentages for demographic factors associated with cirAEs. **(B)** Odds ratios and percentages for clinical features associated with cirAEs. **(C)** Odds ratios and percentages for biomarkers associated with cirAEs. CirAEs, cutaneous immune-related adverse events. BP, bullous pemphigoid. BMI, body mass index. CTLA-4, cytotoxic T lymphocyte antigen-4. PD-1/PD-L1, programmed cell death-1/ligand-1. RCCEP, reactive cutaneous capillary endothelial proliferation. SJS, Stevens-Johnson syndrome. TVEC, talimogene laherparepvec. NSCLC, non-small cell lung cancer. ALC, absolute lymphocyte count. NLR, neutrophil-to-lymphocyte ratio. AEC, absolute eosinophil count. PLR, platelet-to-lymphocyte Ratio. ICIs, immune checkpoint inhibitors. -, Not mentioned in included study.

The odds ratio (OR) for 60 risk factors was reported, including age, body mass index (BMI), smoking history, etc. This section reports risk factors that were found to be statistically significant (*p* < 0.05) and for which ORs with 95% confidence intervals (CIs) were explicitly provided (**Figure 2**).

Demographic factors. Patients aged ≥70 years had a 2.48-fold (95% CI 1.74–3.54) higher risk of overall cirAEs and a 2.32-fold (95% CI 1.19–4.59) increased risk of BP. Patients aged ≥65 years had a 2.77-fold (95% CI 1.36–5.66) higher risk of maculopapular rash. Female sex was associated with overall cirAEs (OR 2.11, 95% CI 1.17–3.82), with postmenopausal women being separately reported as a risk factor for cirAEs (OR 2.17, 95% CI 1.00–4.69). A BMI >25 was associated with overall cirAEs. Overweight (BMI 25–29.9) conferred an OR of 24.7 (95% CI 20.2–30.0), and obesity (BMI ≥30) conferred an OR of 17.7 (95% CI 11.2–26.5). Smoking history was linked to maculopapular rash, with ORs ranging from 1.87 (95% CI 1.02–3.43) to 2.75 (95% CI 1.59–4.75). A smoking index >400 was associated with a high risk of cirAEs (OR 4.35, 95% CI 1.11–16.67).

Clinical features. A history of three or more drug allergies was associated with overall cirAEs (OR 6.57, 95% CI 1.77–24.42). A history of anaphylaxis was associated with pruritus (OR 6.81, 95% CI 1.33–34.67). Pre-existing pemphigus conferred a markedly increased risk of overall cirAEs (OR 35.2, 95% CI 4.08–304). Anti−CTLA−4 therapy increased the risk of pruritus compared with anti−PD−1/L1 therapy (OR 1.83, 95% CI 1.51–2.21). Combination ICI therapy (anti−PD−1/L1 + anti−CTLA−4) was associated with an increased risk of maculopapular rash (OR 2.27, 95% CI 1.70–3.02) and pruritus (OR 1.60, 95% CI 1.27–2.01) compared with monotherapy. Camrelizumab use was associated with RCCEP (OR 8.40, 95% CI 5.15–13.72). Patients with melanoma had a 3.63-fold (95% CI 1.23–10.7) higher likelihood of maculopapular rash and a 91-fold (95% CI 9.80–852) increased risk of vitiligo compared with NSCLC patients. Non−use of aspirin was associated with maculopapular rash (OR 1.47, 95% CI 1.22–1.79), SJS (OR 5.56, 95% CI 1.79–16.67), and vitiligo (OR 2.86, 95% CI 1.05–7.69).

Biomarkers. A higher absolute eosinophil count (AEC >0.205 × 10^9^/L) increased the risk of overall cirAEs (OR 6.14, 95% CI 1.81–20.83). An eosinophil percentage >1.75% was associated with a 4.48-fold increased risk (95% CI 1.14–17.65). A low neutrophil−to−lymphocyte ratio (NLR <2.86) was associated with overall cirAEs (OR 2.69, 95% CI 1.21–6.01). Higher baseline anti−BP180 IgG levels were significantly associated with BP (OR 4.05, 95% CI 1.12–14.66). The presence of HLA−DRB1*11:01 was linked to pruritus (OR 4.53).

## 4 Discussion

In this scoping review, we identified a comprehensive catalog of studies presenting data on various risk factors associated with different cirAEs. Fifty studies were designed as case-control or cohort studies, covering a total of 198,514 patients receiving ICIs, with 24,814 patients (12.5%) experiencing at least one or more cirAEs. Across these studies, 68 risk factors were identified, inductively categorized into 12 domains, and further integrated into 3 overarching areas. Maculopapular rash, pruritus, and unspecified rash were the most frequently reported subtypes, and most documented events were grade 1–2, indicating that cirAEs are common and usually clinically manageable toxicities of ICIs therapy. CirAEs emerged as early as the second day after treatment, with a median time of 23 to 212 days. The wide variation in onset time, phenotype, and effect size across studies suggests that cirAEs do not arise through a single uniform mechanism, but rather reflect the combined influence of host immune background, treatment-related immune activation, tumor-specific biology, and baseline inflammatory status.

A central finding of the present review is that many reported risk factors converge on a similar biological theme, namely a host milieu that is either more immune-reactive or less immunologically restrained at baseline. Older age is one example. Age ≥70 years was associated with overall cirAEs and BP (OR 2.48, 95% CI 1.74–3.54; OR 2.32, 95% CI 1.19–4.59), while age ≥65 years was associated with maculopapular rash (OR 2.77, 95% CI 1.36–5.66). In the corresponding cohorts, these age strata accounted for a substantial proportion of patients, indicating that this is not a rare exposure but a clinically relevant characteristic. Rather than reflecting immune decline alone, aging may predispose to cirAEs through immunosenescence-associated dysregulation, chronic low-grade inflammation, and a greater tendency toward autoimmune phenomena (65–68). Thus, the relationship between age and cirAEs may be better understood as one of altered immune balance rather than simple frailty. However, some studies have not found a significant association between age and cirAEs, but have identified a significant association with other irAEs. It may reflect differences in cohort composition, cancer types, or ICI regimens across studies (69, 70). In any case, advanced age, particularly for patients over 65, is a risk factor that requires special attention in patients receiving immunotherapy.

Sex-related findings point in a similar direction, although their effects appear to be phenotype-dependent. Female sex was associated with overall cirAEs (OR 2.11, 95% CI 1.17–3.82), and postmenopausal women also remained at increased risk (OR 2.17, 95% CI 1.00–4.69). Female sex was additionally linked to maculopapular rash in the included literature. These data are biologically plausible given that females generally mount stronger humoral and cell-mediated immune responses, in part through sex hormone-related immune modulation (71, 72). Conversely, male sex was associated with cirAEs, suggesting that sex may influence not simply the overall frequency of cirAEs, but also the type of cutaneous phenotype that emerges. This distinction is important, because it implies that inflammatory eruptions, pigmentary disorders, and blistering disease may not share a completely identical immunopathological basis.

Obesity and smoking further support the importance of pre-existing inflammatory tone. In the Cortellini cohort (28), overweight and obesity were associated with markedly increased cirAEs risk (OR 24.70, 95% CI 20.20–30.00; OR 17.70, 95% CI 11.20–26.50). Although these effect sizes are striking and should be interpreted cautiously, the direction of the association is biologically credible. Adipose tissue is now recognized as an active immunometabolic organ capable of sustaining chronic cytokine production and altering immune-cell composition, which may lower the threshold for immune-related toxicity once checkpoint inhibition is initiated (73). Smoking showed a similar pattern. Smoking history was associated with cirAEs or maculopapular rash in several studies (OR 2.75, 95% CI 1.59–4.75; OR 1.87, 95% CI 1.02–3.43), and higher cumulative exposure further increased risk (smoking index >400: OR 4.35, 95% CI 1.11–16.67). Rather than functioning merely as a lifestyle variable, smoking may reflect long-term inflammatory conditioning and epigenetic remodeling of immune responses, thereby predisposing patients to exaggerated cutaneous immune activation after ICI exposure (59).

Tumor biology itself also appears to shape cirAEs susceptibility, particularly when tumor antigens overlap with normal skin structures. Melanoma was a particularly relevant example in this review. Compared with NSCLC, melanoma was associated with a higher risk of maculopapular rash (OR 3.63, 95% CI 1.23–10.7), vitiligo (OR 91, 95% CI 9.80–852), and BP (OR 3.21, 95% CI 1.15–6.58). This pattern strongly supports a shared-antigen model, in which antitumor immune responses directed against melanocytic antigens also target normal melanocytes (74, 75). This suggests that, vitiligo may represent a visible marker of antigen-specific collateral damage rather than a nonspecific inflammatory eruption. A similar interpretation may apply to BP-related findings. non−melanoma skin cancer and melanoma were both associated with BP, while higher anti-BP180 IgG levels were also associated with BP. These observations raise the possibility that checkpoint blockade may unmask or amplify pre-existing autoreactivity against structural skin antigens in susceptible patients (97).

Treatment-related factors provide additional evidence that cirAEs risk rises with the breadth and intensity of immune activation. Compared with anti-PD-1/L1 therapy, anti-CTLA-4 therapy increased the risk of pruritus (OR1.83, 95% CI 1.51-2.21), as well as combination anti-PD-1/L1 plus anti-CTLA-4 therapy increased the risk of both phenotypes (OR 2.27, 95% CI 1.70–3.02). These findings are consistent with the broader understanding that more extensive checkpoint release amplifies antitumor immunity but also increases off-target immune toxicity (76). Combination with chemotherapy may exert a similar amplifying effect. In the included studies, ICIs plus chemotherapy were associated with pruritus (OR 6.67, 95% CI 1.30–33.33), possibly because chemotherapy-induced immunogenic cell death increases antigen release and enhances downstream immune activation (77–79). By contrast, camrelizumab-associated RCCEP appears to represent a more drug-specific toxicity. Camrelizumab was strongly associated with RCCEP (OR 8.40, 95% CI 5.15-13.72), indicating that not all cirAEs arise through the same biological pathway. Some may reflect generalized immune amplification, whereas others may involve distinct pharmacologic or vascular mechanisms.

Another major theme in this review is that pre-existing allergic, inflammatory, or autoimmune traits define a clinically high-risk subgroup. Histories of multiple drug allergies and anaphylaxis were associated with overall cirAEs and pruritus (OR 6.57, 95% CI 1.77–24.42; OR 6.81, 95% CI 1.33–34.67). Pre-existing atopic dermatitis, psoriasis, morphea, and pemphigus were also associated with increased cirAEs risk. Although some of these exposures were uncommon within individual cohorts (for example only 2.2% of all patients have a history of penicillin allergy, while only 3.8% have a history of eczema in the included studies), their repeated appearance across studies supports a coherent biological pattern. Patients with an already dysregulated or hyperresponsive immune background may require less additional stimulation for ICIs to precipitate clinically apparent tissue injury (41, 80). Chronic kidney disease stage 3–4 showed a similar association (OR 3.52, 95% CI 2.33–5.31), possibly reflecting chronic systemic inflammation and altered immune homeostasis (41).

Among circulating biomarkers, eosinophil-related measures and lymphocyte-based ratios were repeatedly reported because they may capture baseline immune state before treatment. Higher absolute eosinophil count and eosinophil percentage were associated with cirAEs or RCCEP (OR 6.14, 95% CI 1.81–20.83; OR 4.48, 95% CI 1.14–17.65). Low NLR, low PLR, and low baseline CD4+/CD8+ ratio were likewise associated with increased toxicity (OR 2.69, 95% CI 1.21–6.01; OR 6.15, 95% CI 1.67–22.56; OR 3.27, 95% CI 1.43–7.46). All of these biomarker measures indicate that cirAEs tend to occur in patients whose baseline immune balance has shifted toward a reactive state rather than a suppressive one. Eosinophils may indicate an allergic or type 2 inflammatory tendency, whereas low NLR or PLR may reflect a less immunosuppressive, more lymphocyte-permissive immune environment that facilitates both antitumor activity and immune-related toxicity (81–83). Cytokines, including higher levels of GM-CSF, CCL19, and IL-4, can enhance immune effects (84, 85). The associations of HLA-DRB1*11:01 with pruritus (OR 4.53) and anti-BP180 IgG with BP further support the idea that inherited immune presentation patterns and baseline autoreactivity may influence not only whether toxicity occurs, but also which phenotype emerges (86).

One intriguing finding was the association between non-use of aspirin and several cirAEs subtypes, including maculopapular rash, SJS, and vitiligo (OR 1.47, 95% CI 1.22–1.79; OR 5.56, 95% CI 1.79–16.67; OR 2.86, 95% CI 1.05–7.69). One potential mechanism underlying aspirin’s protective effect may be related to its acetylating action on histones. This action leads to chromatin remodeling, which reduces the transcription of pro-inflammatory cytokine genes in immune cells, such as macrophages and T cells. Consequently, it can inhibit the secretion of pro-inflammatory cytokines by immune cells and mitigate excessive immune responses (87, 88). Nevertheless, since this signal arose from observational pharmacovigilance-based evidence, it should be interpreted cautiously and cannot be taken as proof of a protective aspirin effect. Some studies have shown that aspirin can promote T cell activation, thereby increasing irAEs (89–92). Existing studies have not analyzed the reasons for these contradictory results. Therefore, this observation should be regarded as hypothesis-generating rather than practice-changing.

Not all factors identified in the Results are discussed here in equal detail. We prioritized variables that were repeatedly reported, had interpretable ORs, or fit a biologically coherent framework, whereas factors reported only once or lacking clear effect estimates were not expanded further. Overall, the most interpretable patterns involved host immune reactivity and chronic inflammatory background, tumor-specific or shared-antigen biology, and treatment intensity together with biomarker-defined immune context.

To our knowledge, this is the first and most comprehensive assessment of risk factors for cirAEs to date, with the scope to identify nearly all relevant studies. However, due to feasibility considerations, we limited the search terms to titles and abstracts, which may result in missing some studies that mention relevant content in the full text. Additionally, to ensure comprehensive information acquisition, we excluded articles for which the full text was not available, which may result in missing some new findings. Articles in languages other than Chinese and English were not included, but this had a very limited impact on the results. Furthermore, we searched 9 databases and manually reviewed the references of relevant articles, leading us to believe that the resulting data have reached saturation.

## 5 Conclusion

Overall, this scoping review offers a thorough analysis of a wide array of potential risk factors for cirAEs in cancer patients treated with immune checkpoint inhibitors. The review identified numerous demographic, clinical, and biomarker-related risk factors that contribute to the development of cirAEs. However, our understanding of how these factors interact and influence the immune response leading to cirAEs remains incomplete. Further research is essential to deepen our knowledge of the underlying mechanisms and to develop effective preventative strategies. Additionally, it is crucial to enhance the quality of research by encouraging adherence to standardized reporting guidelines for immunotherapy-related adverse events. This will facilitate the identification of clinically valuable risk factors and improve the management of patients undergoing immunotherapy.

## Data Availability

The original contributions presented in the study are included in the article/**Supplementary Material**. Further inquiries can be directed to the corresponding author.
