# Supplementary material for: Risk factors for cutaneous immune-related adverse events: a systematic scoping review
Source: Front Immunol. 2026 Apr 16;17:1722781. doi: 10.3389/fimmu.2026.1722781 (PMC13128564; doi:10.3389/fimmu.2026.1722781)
Supplement: Supplementary file 2 [file Table2.docx]

**Appendix 1** Search Terms used to find studies

Search started on Dec. 31^st^, 2023 with no time restrictions.

[Database: Pubmed 2](#_Toc196665206)

[Database: Embase 4](#_Toc196665207)

[Database: Cochrane 5](#_Toc196665208)

[Database: Web of science 7](#_Toc196665209)

[Database: ProQuest 9](#_Toc196665210)

[Database: CINAHL 10](#_Toc196665211)

[Databases: Chinese databases, including CNKI, Wanfang Data, VIP and SinoMed 11](#_Toc196665212)

# Database: Pubmed

|  | Searches | Results |
| --- | --- | --- |
| #1 | "Neoplasms"[MeSH] OR "cancer"[Title/Abstract] OR "neoplas*"[Title/Abstract] OR "carcinoma"[Title/Abstract] OR "tumor"[Title/Abstract] OR "tumour"[Title/Abstract] OR "adenocarcinoma"[Title/Abstract] OR "malignan*"[Title/Abstract] | 5,117,855 |
| #2 | "Immunotherapy"[Mesh] OR "Immune Checkpoint Inhibitors"[Mesh] OR "Immune Checkpoint Blockers"[Title/Abstract] OR "Ipilimumab"[Title/Abstract] OR "Pembrolizumab"[Title/Abstract] OR "Nivolumab"[Title/Abstract] OR "Atezolizumab"[Title/Abstract] OR "Tremelimumab"[Title/Abstract] OR "Avelumab"[Title/Abstract] OR "Durvalumab"[Title/Abstract] OR "Programmed Cell Death 1 Receptor"[Mesh] OR "Programmed Death Ligand 1 Inhibitors"[Title/Abstract] OR "Cytotoxic T-Lymphocyte-Associated Antigen 4"[Title/Abstract] OR "PD-1"[Title/Abstract] OR "PD-L1"[Title/Abstract] OR "CTLA-4"[Title/Abstract] | 396,198 |
| #3 | "Drug-Related Side Effects and Adverse Reactions"[Mesh] OR "Adverse reactions"[Title/Abstract] OR "Adverse events"[Title/Abstract] OR "Adverse effects"[Title/Abstract] OR "Toxicities"[Title/Abstract] | 573,112 |
| #4 | "Immune-related Adverse events"[Title/Abstract] OR "Immune-related Toxicities"[Title/Abstract] OR "Immune-related Side Effect"[Title/Abstract] OR "irAEs"[Title/Abstract] | 5,804 |
| #5 | "Vitiligo"[Title/Abstract] OR "Morbilliform"[Title/Abstract] OR "Maculopapular"[Title/Abstract] OR "Exanthema"[Mesh] OR "Rash"[Title/Abstract] OR "RCCEP"[Title/Abstract] OR "reactive cutaneous capillary endothelial proliferation"[Title/Abstract] OR "pruritus"[MeSH Terms] OR "xerosis"[Title/Abstract] OR "psoriasiform"[Title/Abstract] OR "Grover disease"[Title/Abstract] OR "erythroderma"[Title/Abstract] OR "pemphigoid, bullous"[MeSH Terms] OR "bullous"[Title/Abstract] OR "lichenoid"[Title/Abstract] OR "acneiform"[Title/Abstract] OR "papulopustular"[Title/Abstract] OR "granulomatous"[Title/Abstract] OR "alopecia"[MeSH Terms] OR "pityriasis rubra pilaris"[MeSH Terms] OR "scleroderma*"[Title/Abstract] OR "mucositis"[MeSH Terms] OR "pityriasis rosea"[Title/Abstract] OR "Stevens-Johnson Syndrome"[MeSH Terms] OR "toxic epidermal necrolysis"[Title/Abstract] OR "SJS"[Title/Abstract] OR "TEN"[Title/Abstract] OR "drug reaction with eosinophilia and systemic symptoms"[Title/Abstract] OR "Drug Hypersensitivity Syndrome"[MeSH Terms] OR "DRESS"[Title/Abstract] OR "DIHS"[Title/Abstract] OR "Acute Generalized Exanthematous Pustulosis"[MeSH Terms] OR "AGEP"[Title/Abstract] OR "reactive cutaneous capillary endothelial proliferation"[Title/Abstract] | 532,230 |
| #6 | "Cutaneous"[Title/Abstract] | 180,370 |
| #7 | "cirAE"[Title/Abstract] OR "cutaneous immune-related adverse event"[Title/Abstract] "cirAEs"[Title/Abstract] OR "cutaneous immune-related adverse events"[Title/Abstract] | 100 |
| #8 | ("Risk Factors"[Mesh] OR "Factor, Risk"[Title/Abstract] OR "Risk Factor"[Title/Abstract] OR "Social Risk Factors"[Title/Abstract] OR "Risk Factors, Social"[Title/Abstract] OR "Health Correlates"[Title/Abstract] OR "Correlates, Health"[Title/Abstract] OR "Population at Risk"[Title/Abstract] OR "Populations at Risk"[Title/Abstract] OR "Risk Scores"[Title/Abstract] OR "Risk Score"[Title/Abstract] OR "Risk Factor Scores"[Title/Abstract] OR "Risk Factor Score"[Title/Abstract]) OR ("Influence Factor"[Title/Abstract] OR "Influence Factors"[Title/Abstract] OR "Biological Factors"[Mesh] OR "Biological Factor"[Title/Abstract] OR "Sociological Factors"[Mesh] OR "Sociological Factor"[Title/Abstract] OR "Sociological Phenomena"[Title/Abstract] OR "Social Characteristics"[Title/Abstract] OR "Social Attributes"[Title/Abstract] OR "Social Attribute"[Title/Abstract] OR "Epidemiologic Factors"[Mesh] OR "Determinants, Epidemiologic"[Title/Abstract] OR "Epidemiologic Determinants"[Title/Abstract] OR "Factor, Epidemiologic"[Title/Abstract] OR "Factors, Epidemiologic"[Title/Abstract] OR "Epidemiologic Factor"[Title/Abstract]) | 6,244,819 |
| #9 | ((#1 AND #2 AND #3) OR #4) AND (#5 OR #6 OR #7) AND #8 | 577 |

# Database: Embase

|  | Researches | Results |
| --- | --- | --- |
| #1 | 'malignant neoplasms' OR 'cancer':ti,ab,kw OR 'neoplas*':ti,ab,kw OR 'carcinoma':ti,ab,kw OR 'tumor':ti,ab,kw OR 'tumour':ti,ab,kw OR 'adenocarcinoma':ti,ab,kw OR 'malignan*':ti,ab,kw | 5,435,260 |
| #2 | 'immunotherapy'/exp OR 'immune checkpoint inhibitor'/exp OR 'immune therapy':ti,ab,kw OR 'immune checkpoint blocker':ti,ab,kw OR 'ipilimumab':ti,ab,kw OR 'pembrolizumab':ti,ab,kw OR 'nivolumab':ti,ab,kw OR 'atezolizumab':ti,ab,kw OR 'tremelimumab':ti,ab,kw OR 'avelumab':ti,ab,kw OR 'durvalumab':ti,ab,kw OR 'programmed death 1 receptor'/exp OR 'programmed death ligand 1 inhibitors':ti,ab,kw OR 'cytotoxic t-lymphocyte-associated antigen 4':ti,ab,kw OR 'pd-1':ti,ab,kw OR 'pd-l1':ti,ab,kw OR 'ctla-4':ti,ab,kw | 445,849 |
| #3 | 'adverse drug reaction'/exp OR 'adverse reactions' OR 'adverse event*':ti,ab,kw OR 'adverse effect*':ti,ab,kw OR 'toxicities':ti,ab,kw | 1,308,213 |
| #4 | 'immune-related adverse events':ti,ab,kw OR 'immune-related toxicities':ti,ab,kw OR 'immune-related side effect':ti,ab,kw OR 'iraes':ti,ab,kw | 9,984 |
| #5 | 'vitiligo'/exp OR 'morbilliform':ti,ab,kw OR 'maculopapular rash'/exp OR 'maculopapular':ti,ab,kw OR 'maculo-papular eruption':ti,ab,kw OR 'maculo-papular eruptions':ti,ab,kw OR 'maculopapular rashes':ti,ab,kw OR 'maculopapular rash':ti,ab,kw OR ‘rash’/exp OR 'reactive cutaneous capillary endothelial proliferation':ti,ab,kw OR 'rccep':ti,ab,kw OR 'pruritus'/exp OR 'xerosis'/exp OR 'psoriasiform':ti,ab,kw OR 'transient acantholytic dermatosis'/exp OR 'grover disease':ti,ab,kw OR 'exanthema':ti,ab,kw OR 'bullous pemphigoid'/exp OR 'bullous':ti,ab,kw OR 'lichenoid'/exp OR 'acneiform':ti,ab,kw OR 'papulopustular':ti,ab,kw OR 'granulomatous':ti,ab,kw OR 'alopecia'/exp OR 'pityriasis rubra pilaris'/exp OR 'scleroderma*':ti,ab,kw OR 'mucositis':ti,ab,kw OR 'pityriasis rosea'/exp OR 'stevens johnson syndrome'/exp OR 'toxic epidermal necrolysis'/exp OR 'stevens johnson/toxic epidermal necrolysis overlap syndrome'/exp OR 'sjs':ti,ab,kw OR 'ten':ti,ab,kw OR 'drug reaction with eosinophilia and systemic symptoms':ti,ab,kw OR 'drug hypersensitivity syndrome':ti,ab,kw OR 'dress syndrome'/exp OR 'dihs':ti,ab,kw OR 'acute generalized exanthematous pustulosis'/exp OR 'agep':ti,ab,kw | 946,501 |
| #6 | 'cutaneous':ti,ab,kw | 250,088 |
| #7 | 'cirAE':ti,ab,kw OR 'cutaneous immune-related adverse event':ti,ab,kw OR 'cirAEs':ti,ab,kw OR 'cutaneous immune-related adverse events':ti,ab,kw | 156 |
| #8 | 'risk factor'/exp OR 'relative risk':ti,ab,kw OR 'risk factors':ti,ab,kw OR 'risk factor':ti,ab,kw OR 'influence factors':ti,ab,kw OR 'influence factor':ti,ab,kw OR 'biological factor'/exp OR 'biological factors':ti,ab,kw OR 'social aspects and related phenomena'/exp OR 'sociological factors':ti,ab,kw OR 'epidemiologic factors':ti,ab,kw OR 'epidemiologic factor':ti,ab,kw | 8,769,189 |
| #9 | ((#1 AND #2 AND #3) OR #4) AND (#5 OR #6 OR #7) AND #8 | 3,353 |

# Database: Cochrane

|  | Searches | Results |
| --- | --- | --- |
| #1 | (neoplasms)/exp OR (cancer):ti,ab,kw OR (neoplas*):ti,ab,kw OR (carcinoma):ti,ab,kw OR (tumor):ti,ab,kw OR (tumour):ti,ab,kw OR (adenocarcinoma):ti,ab,kw OR (malignan*):ti,ab,kw | 273,079 |
| #2 | (Immunotherapy)/exp OR (Immune Checkpoint Inhibitors)/exp OR (immune checkpoint block*):ti,ab,kw OR (Ipilimumab):ti,ab,kw OR (Pembrolizumab):ti,ab,kw OR (Nivolumab):ti,ab,kw OR (Atezolizumab):ti,ab,kw OR (Tremelimumab):ti,ab,kw OR (Avelumab):ti,ab,kw OR (Durvalumab):ti,ab,kw OR (Programmed Death 1 Receptor):ti,ab,kw OR (Programmed Death Ligand 1 Inhibitors):ti,ab,kw OR (Cytotoxic T-Lymphocyte-Associated Antigen 4):ti,ab,kw OR (PD-1):ti,ab,kw OR (PD-L1):ti,ab,kw OR (CTLA-4):ti,ab,kw | 21,882 |
| #3 | (Drug-Related Side Effects and Adverse Reactions)/exp OR (Adverse reactions):ti,ab,kw OR (Adverse event*):ti,ab,kw OR (Adverse effect*):ti,ab,kw OR (toxicities):ti,ab,kw | 363,159 |
| #4 | (Immune-related Adverse events):ti,ab,kw OR (Immune-related Toxicities):ti,ab,kw OR (Immune-related Side Effect):ti,ab,kw OR (irAEs):ti,ab,kw | 617 |
| #5 | (Vitiligo):ti,ab,kw OR (Morbilliform):ti,ab,kw OR (Maculopapular):ti,ab,kw OR (Exanthema)/exp OR (Rash):ti,ab,kw OR (RCCEP):ti,ab,kw OR (reactive cutaneous capillary endothelial proliferation):ti,ab,kw OR (pruritus)/exp OR (xerosis):ti,ab,kw OR (psoriasiform):ti,ab,kw OR (Grover disease):ti,ab,kw OR (erythroderma):ti,ab,kw OR (pemphigoid, bullous)/exp OR (bullous):ti,ab,kw OR (lichenoid):ti,ab,kw OR (acneiform):ti,ab,kw OR (papulopustular):ti,ab,kw OR (granulomatous):ti,ab,kw OR (alopecia)/exp OR (pityriasis rubra pilaris)/exp OR (scleroderma*):ti,ab,kw OR (mucositis)/exp OR (pityriasis rosea):ti,ab,kw OR (Stevens-Johnson Syndrome)/exp OR (toxic epidermal necrolysis):ti,ab,kw OR (SJS):ti,ab,kw OR (TEN):ti,ab,kw OR (drug reaction with eosinophilia and systemic symptoms):ti,ab,kw OR (Drug Hypersensitivity Syndrome)/exp OR (DRESS):ti,ab,kw OR (DIHS):ti,ab,kw OR (Acute Generalized Exanthematous Pustulosis)/exp OR (AGEP):ti,ab,kw OR (reactive cutaneous capillary endothelial proliferation):ti,ab,kw | 50,771 |
| #6 | (Cutaneous):ti,ab,kw | 14,317 |
| #7 | (cirAE):ti,ab,kw OR (cutaneous immune-related adverse event):ti,ab,kw (cirAEs):ti,ab,kw OR (cutaneous immune-related adverse events):ti,ab,kw | 30 |
| #8 | ((Risk Factors)/exp OR (Factor, Risk):ti,ab,kw OR (Risk Factor):ti,ab,kw OR (Social Risk Factors):ti,ab,kw OR (Risk Factors, Social):ti,ab,kw OR (Health Correlates):ti,ab,kw OR (Correlates, Health):ti,ab,kw OR (Population at Risk):ti,ab,kw OR (Populations at Risk):ti,ab,kw OR (Risk Scores):ti,ab,kw OR (Risk Score):ti,ab,kw OR (Risk Factor Scores):ti,ab,kw OR (Risk Factor Score):ti,ab,kw) OR ((Influence Factor):ti,ab,kw OR (Influence Factors):ti,ab,kw OR (Biological Factors)/exp OR (Biological Factor):ti,ab,kw OR (Sociological Factors)/exp OR (Sociological Factor):ti,ab,kw OR (Sociological Phenomena):ti,ab,kw OR (Social Characteristics):ti,ab,kw OR (Social Attributes):ti,ab,kw OR (Social Attribute):ti,ab,kw OR (Epidemiologic Factors)/exp OR (Determinants, Epidemiologic):ti,ab,kw OR (Epidemiologic Determinants):ti,ab,kw OR (Factor, Epidemiologic):ti,ab,kw OR (Factors, Epidemiologic):ti,ab,kw OR (Epidemiologic Factor):ti,ab,kw) | 301,888 |
| #9 | ((#1 AND #2 AND #3) OR #4)) AND (#5 OR #6 OR #7) AND #8 | 217 |

# Database: Web of science

|  | Researches | Results |
| --- | --- | --- |
| #1 | TI=(Neoplasm) OR TI=(cancer) OR TI=(neoplas*) OR TI=(carcinoma) OR TI=(tumor) OR TI=(tumour) OR TI=(adenocarcinoma) OR TI=(malignan*) OR AB=(Neoplasm) OR AB=(cancer) OR AB=(neoplas*) OR AB=(carcinoma) OR AB=(tumor) OR AB=(tumour) OR AB=(adenocarcinoma) OR AB=(malignan*) | 6,539,403 |
| #2 | TI=(Immunotherapy) OR TI=(Immune Checkpoint Inhibitors) OR TI=(Immune Checkpoint Blockers) OR TI=(Ipilimumab) OR TI=(Pembrolizumab) OR TI=(Nivolumab) OR TI=(Atezolizumab) OR TI=(Tremelimumab) OR TI=(Avelumab) OR TI=(Durvalumab) OR TI=(Programmed Cell Death 1 Receptor) OR TI=(Programmed Death Ligand 1 Inhibitors) OR TI=(Cytotoxic T-Lymphocyte-Associated Antigen 4) OR TI=(PD-1) OR TI=(PD-L1) OR TI=(CTLA-4) OR AB=(Immunotherapy) OR AB=(Immune Checkpoint Inhibitors) OR AB=(Immune Checkpoint Blockers) OR AB=(Ipilimumab) OR AB=(Pembrolizumab) OR AB=(Nivolumab) OR AB=(Atezolizumab) OR AB=(Tremelimumab) OR AB=(Avelumab) OR AB=(Durvalumab) OR AB=(Programmed Cell Death 1 Receptor) OR AB=(Programmed Death Ligand 1 Inhibitors) OR AB=(Cytotoxic T-Lymphocyte-Associated Antigen 4) OR AB=(PD-1) OR AB=(PD-L1) OR AB=(CTLA-4) | 289,252 |
| #3 | TI=(Drug-Related Side Effects and Adverse Reactions) OR TI=(Adverse reactions) OR TI=(Adverse events) OR TI=(Adverse effects) OR TI=(Toxicities) OR AB=(Drug-Related Side Effects and Adverse Reactions) OR AB=(Adverse reactions) OR AB=(Adverse events) OR AB=(Adverse effects) OR AB=(Toxicities) | 1,751,353 |
| #4 | TI=(Immune-related Adverse events) OR TI=(Immune-related Toxicities) OR TI=(Immune-related Side Effect) OR TI=(irAEs) OR AB=(Immune-related Adverse events) OR AB=(Immune-related Toxicities) OR AB=(Immune-related Side Effect) OR AB=(irAEs) | 9,033 |
| #5 | TI=(Vitiligo) OR TI=(Morbilliform) OR TI=(Maculopapular) OR TI=(Exanthema) OR TI=(Rash) OR TI=(RCCEP) OR TI=(reactive cutaneous capillary endothelial proliferation) OR TI=(pruritus) OR TI=(xerosis) OR TI=(psoriasiform) OR TI=(Grover disease) OR TI=(erythroderma) OR TI=(pemphigoid, bullous) OR TI=(bullous) OR TI=(lichenoid) OR TI=(acneiform) OR TI=(papulopustular) OR TI=(granulomatous) OR TI=(alopecia) OR TI=(pityriasis rubra pilaris) OR TI=(scleroderma*) OR TI=(mucositis) OR TI=(pityriasis rosea) OR TI=(Stevens-Johnson Syndrome) OR TI=(toxic epidermal necrolysis) OR TI=(SJS) OR TI=(TEN) OR TI=(drug reaction with eosinophilia and systemic symptoms) OR TI=(Drug Hypersensitivity Syndrome) OR TI=(DRESS) OR TI=(DIHS) OR TI=(Acute Generalized Exanthematous Pustulosis) OR TI=(AGEP) OR TI=(reactive cutaneous capillary endothelial proliferation) OR AB=(Vitiligo) OR AB=(Morbilliform) OR AB=(Maculopapular) OR AB=(Exanthema) OR AB=(Rash) OR AB=(RCCEP) OR AB=(reactive cutaneous capillary endothelial proliferation) OR AB=(pruritus) OR AB=(xerosis) OR AB=(psoriasiform) OR AB=(Grover disease) OR AB=(erythroderma) OR AB=(pemphigoid, bullous) OR AB=(bullous) OR AB=(lichenoid) OR AB=(acneiform) OR AB=(papulopustular) OR AB=(granulomatous) OR AB=(alopecia) OR AB=(pityriasis rubra pilaris) OR AB=(scleroderma*) OR AB=(mucositis) OR AB=(pityriasis rosea) OR AB=(Stevens-Johnson Syndrome) OR AB=(toxic epidermal necrolysis) OR AB=(SJS) OR AB=(TEN) OR AB=(drug reaction with eosinophilia and systemic symptoms) OR AB=(Drug Hypersensitivity Syndrome) OR AB=(DRESS) OR AB=(DIHS) OR AB=(Acute Generalized Exanthematous Pustulosis) OR AB=(AGEP) OR AB=(reactive cutaneous capillary endothelial proliferation) | 1,289,706 |
| #6 | TI=(Cutaneous) OR AB=(Cutaneous) | 255,014 |
| #7 | TI=(cirAE) OR TI=(cutaneous immune-related adverse event) OR TI=(cirAEs) OR TI=(cutaneous immune-related adverse events) OR AB=(cirAE) OR AB=(cutaneous immune-related adverse event) OR AB=(cirAEs) OR AB=(cutaneous immune-related adverse events) | 440 |
| #8 | (TI=(Risk Factors) OR TI=(Factor, Risk) OR TI=(Risk Factor) OR TI=(Social Risk Factors) OR TI=(Risk Factors, Social) OR TI=(Health Correlates) OR TI=(Correlates, Health) OR TI=(Population at Risk) OR TI=(Populations at Risk) OR TI=(Risk Scores) OR TI=(Risk Score) OR TI=(Risk Factor Scores) OR TI=(Risk Factor Score)) OR (TI=(Influence Factor) OR TI=(Influence Factors) OR TI=(Biological Factors) OR TI=(Biological Factor) OR TI=(Sociological Factors) OR TI=(Sociological Factor) OR TI=(Sociological Phenomena) OR TI=(Social Characteristics) OR TI=(Social Attributes) OR TI=(Social Attribute) OR TI=(Epidemiologic Factors) OR TI=(Determinants, Epidemiologic) OR TI=(Epidemiologic Determinants) OR TI=(Factor, Epidemiologic) OR TI=(Factors, Epidemiologic) OR TI=(Epidemiologic Factor)) OR (AB=(Risk Factors) OR AB=(Factor, Risk) OR AB=(Risk Factor) OR AB=(Social Risk Factors) OR AB=(Risk Factors, Social) OR AB=(Health Correlates) OR AB=(Correlates, Health) OR AB=(Population at Risk) OR AB=(Populations at Risk) OR AB=(Risk Scores) OR AB=(Risk Score) OR AB=(Risk Factor Scores) OR AB=(Risk Factor Score)) OR (AB=(Influence Factor) OR AB=(Influence Factors) OR AB=(Biological Factors) OR AB=(Biological Factor) OR AB=(Sociological Factors) OR AB=(Sociological Factor) OR AB=(Sociological Phenomena) OR AB=(Social Characteristics) OR AB=(Social Attributes) OR AB=(Social Attribute) OR AB=(Epidemiologic Factors) OR AB=(Determinants, Epidemiologic) OR AB=(Epidemiologic Determinants) OR AB=(Factor, Epidemiologic) OR AB=(Factors, Epidemiologic) OR AB=(Epidemiologic Factor)) | 4,226,053 |
| #9 | ((#1 AND #2 AND #3) OR #4) AND (#5 OR #6 OR #7) AND #8 | 201 |

# Database: ProQuest

|  | Searches | Results |
| --- | --- | --- |
| S1 | (SU(malignant neoplasms) OR TI,AB(cancer) OR TI,AB(neoplas*) OR TI,AB(carcinoma) OR TI,AB(tumor) OR TI,AB(tumour) OR TI,AB(adenocarcinoma) OR TI,AB(malignan*)) AND (SU(Immunotherapy) OR SU(Immune Checkpoint Inhibitor) OR TI,AB(Immune Checkpoint Blockers) OR TI,AB(Ipilimumab) OR TI,AB(Pembrolizumab) OR TI,AB(Nivolumab) OR TI,AB(Atezolizumab) OR TI,AB(Tremelimumab) OR TI,AB(Avelumab) OR TI,AB(Durvalumab) OR SU(Programmed Death 1 Receptor) OR TI,AB(Programmed Death Ligand 1 Inhibitors) OR TI,AB(Cytotoxic T-Lymphocyte-Associated Antigen 4) OR TI,AB(PD-1) OR TI,AB(PD-L1) OR TI,AB(CTLA-4)) AND (SU(adverse drug reaction) OR SU(Adverse reactions) OR TI,AB(Adverse event*) OR TI,AB(Adverse effect*) OR TI,AB(Toxicities)) | 13,435 |
| S2 | TI,AB(Immune-related Adverse events) OR TI,AB(Immune-related Toxicities) OR TI,AB(Immune-related Side Effect) OR TI,AB(irAEs) | 3,770 |
| S3 | SU(vitiligo) OR TI,AB(morbilliform) OR SU(maculopapular rash) OR TI,AB(maculopapular) OR TI,AB(maculo-papular eruption) OR TI,AB(maculo-papular eruptions) OR TI,AB(maculopapular rashes) OR TI,AB(maculopapular rash) OR SU(rash) OR TI,AB(reactive cutaneous capillary endothelial proliferation) OR TI,AB(rccep) OR TI,AB(pruritus) OR SU(xerosis) OR TI,AB(psoriasiform) OR SU(transient acantholytic dermatosis) OR TI,AB(grover disease) OR TI,AB(exanthema) OR SU(bullous pemphigoid) OR TI,AB(bullous) OR SU(lichenoid) OR TI,AB(acneiform) OR TI,AB(papulopustular) OR TI,AB(granulomatous) OR SU(alopecia) OR SU(pityriasis rubra pilaris) OR TI,AB(scleroderma*) OR TI,AB(mucositis) OR SU(pityriasis rosea) OR SU(stevens johnson syndrome) OR SU(toxic epidermal necrolysis) OR SU(stevens johnson/toxic epidermal necrolysis overlap syndrome) OR TI,AB(sjs) OR TI,AB(ten) OR TI,AB(drug reaction with eosinophilia and systemic symptoms) OR TI,AB(drug hypersensitivity syndrome) OR SU(dress syndrome) OR TI,AB(dihs) OR SU(acute generalized exanthematous pustulosis) OR TI,AB(agep) | 670,301 |
| S4 | TI,AB(cutaneous) | 61,294 |
| S5 | TI,AB(cirAE) OR TI,AB(cutaneous immune-related adverse event) OR TI,AB(cirAEs) OR TI,AB(cutaneous immune-related adverse events) | 177 |
| S6 | SU(risk factor) OR TI,AB(relative risk) OR TI,AB(risk factors) OR TI,AB(risk factor) OR TI,AB(influence factors) OR TI,AB(influence factor) OR SU(biological factor) OR TI,AB(biological factors) OR SU(social aspects and related phenomena) OR TI,AB(sociological factors) OR TI,AB(epidemiologic factors) OR TI,AB(epidemiologic factor) | 1,542,490 |
| S7 | ([S1] OR [S2]) AND ([S3] OR [S4] OR [S5]) AND [S6] | 108 |

# Database: CINAHL

|  | Searches | Results |
| --- | --- | --- |
| S1 | ((((SU Neoplasm) OR (AB cancer) OR (AB neoplas*) OR (AB carcinoma) OR (AB tumor) OR (AB tumour) OR (AB adenocarcinoma) OR (AB malignan*)) AND ((SU Immunotherapy) OR (AB Immune Checkpoint Inhibitors) OR (AB Immune Checkpoint Blockers) OR (AB Ipilimumab) OR (AB Pembrolizumab) OR (AB Nivolumab) OR (AB Atezolizumab) OR (AB Tremelimumab) OR (AB Avelumab) OR (AB Durvalumab) OR (SU Programmed Cell Death 1 Receptor) OR (AB Programmed Death Ligand 1 Inhibitors) OR (AB Cytotoxic T-Lymphocyte-Associated Antigen 4) OR (AB PD-1) OR (AB PD-L1) OR (AB CTLA-4)) AND ((SU Drug-Related Side Effects and Adverse Reactions) OR (AB Adverse reactions) OR (AB Adverse events) OR (AB Adverse effects) OR (AB Toxicities))) OR ((AB Immune-related Adverse events) OR (AB Immune-related Toxicities) OR (AB Immune-related Side Effect) OR (AB irAEs))) AND (((AB Vitiligo) OR (AB Morbilliform) OR (AB Maculopapular) OR (SU Exanthema) OR (AB Rash) OR (AB RCCEP) OR (AB reactive cutaneous capillary endothelial proliferation) OR (SU pruritus) OR (AB xerosis) OR (AB psoriasiform) OR (AB Grover disease) OR (AB erythroderma) OR (SU pemphigoid, bullous) OR (AB bullous) OR (AB lichenoid) OR (AB acneiform) OR (AB papulopustular) OR (AB granulomatous) OR (SU alopecia) OR (SU pityriasis rubra pilaris) OR (AB scleroderma*) OR (SU mucositis) OR (AB pityriasis rosea) OR (SU Stevens-Johnson Syndrome) OR (AB toxic epidermal necrolysis) OR (AB SJS) OR (AB TEN) OR (AB drug reaction with eosinophilia and systemic symptoms) OR (SU Drug Hypersensitivity Syndrome) OR (AB DRESS) OR (AB DIHS) OR (SU Acute Generalized Exanthematous Pustulosis) OR (AB AGEP) OR (AB reactive cutaneous capillary endothelial proliferation)) OR (AB Cutaneous) OR ((AB cirAE) OR (AB cutaneous immune-related adverse event) (AB cirAEs) OR (AB cutaneous immune-related adverse events))) AND (((SU Risk Factors) OR (AB Factor, Risk) OR (AB Risk Factor) OR (AB Social Risk Factors) OR (AB Risk Factors, Social) OR (AB Health Correlates) OR (AB Correlates, Health) OR (AB Population at Risk) OR (AB Populations at Risk) OR (AB Risk Scores) OR (AB Risk Score) OR (AB Risk Factor Scores) OR (AB Risk Factor Score)) OR ((AB Influence Factor) OR (AB Influence Factors) OR (SU Biological Factors) OR (AB Biological Factor) OR (SU Sociological Factors) OR (AB Sociological Factor) OR (AB Sociological Phenomena) OR (AB Social Characteristics) OR (AB Social Attributes) OR (AB Social Attribute) OR (SU Epidemiologic Factors) OR (AB Determinants, Epidemiologic) OR (AB Epidemiologic Determinants) OR (AB Factor, Epidemiologic) OR (AB Factors, Epidemiologic) OR (AB Epidemiologic Factor))) | 64 |

# Databases: Chinese databases, including CNKI, Wanfang Data, VIP and SinoMed

| Database | Research terms | Results |
| --- | --- | --- |
| CNKI | TKA=('免疫治疗' + '免疫检查点抑制剂' + 'PD-1' + 'PD-L1' + 'CTLA-4') AND TKA=('不良反应' + '不良事件' + '副作用' + '毒副反应') AND TKA=('皮肤反应' + '皮肤毒性' + '皮肤免疫相关不良事件' + '皮疹' + '瘙痒' + '湿疹' + '白癜风' + '硬皮病' + '牛皮藓' + '格罗弗病' + '斑丘疹' + '类天疱疮' + '大疱' + '苔藓样' + '痤疮样' + '丘疹脓疱性' + '肉芽肿' + '麻疹' + '斑秃' + '脱发' + '毛发红糠疹' + '硬皮病' + '黏膜炎' + '玫瑰糠疹' + '史蒂文斯-约翰逊综合征' + 'Stevens-Johnson综合征' + '中毒性表皮坏死松解症' + 'SJS' + 'TEN' + '伴有嗜酸性粒细胞增多和全身症状的药物反应' + '药物过敏综合征' + 'DRESS' + '急性全身性发疹性脓疱病' + 'AGEP' + '皮肤毛细血管增生症' + 'RCCEP') AND TKA=('危险因素' + '影响因素' + '高危因素' + '发病因素') | 103 |
| Wanfang Data | 题名或关键词:("免疫治疗" OR "免疫检查点抑制剂" OR "PD-1" OR "PD-L1" OR "CTLA-4") and 题名或关键词:("不良反应" OR "不良事件" OR "副作用" OR "毒副反应") and 题名或关键词:("皮肤反应" OR "皮肤毒性" OR "皮肤免疫相关不良事件" OR "皮疹" OR "瘙痒" OR "湿疹" OR "白癜风" OR "硬皮病" OR "牛皮藓" OR "格罗弗病" OR "斑丘疹" OR "类天疱疮" OR "大疱" OR "苔藓样" OR "痤疮样" OR "丘疹脓疱性" OR "肉芽肿" OR "麻疹" OR "斑秃" OR "脱发" OR "毛发红糠疹" OR "硬皮病" OR "黏膜炎" OR "玫瑰糠疹" OR "史蒂文斯-约翰逊综合征" OR "Stevens-Johnson综合征" OR "中毒性表皮坏死松解症" OR "SJS" OR "TEN" OR "伴有嗜酸性粒细胞增多和全身症状的药物反应" OR "药物过敏综合征" OR "DRESS" OR "急性全身性发疹性脓疱病" OR "AGEP" OR "皮肤毛细血管增生症" OR "RCCEP") and 题名或关键词:("危险因素" OR "影响因素" OR "高危因素" OR "发病因素") | 8 |
| VIP | (R=(免疫治疗 + 免疫检查点抑制剂 + PD-1 + PD-L1 + CTLA-4)) AND (R=(不良反应 + 不良事件 + 副作用 + 毒副反应)) AND (R=(皮肤反应 + 皮肤毒性 + 皮肤免疫相关不良事件 + 皮疹 + 瘙痒 + 湿疹 + 白癜风 + 硬皮病 + 牛皮藓 + 格罗弗病 + 斑丘疹 + 类天疱疮 + 大疱 + 苔藓样 + 痤疮样 + 丘疹脓疱性 + 肉芽肿 + 麻疹 + 斑秃 + 脱发 + 毛发红糠疹 + 硬皮病 + 黏膜炎 + 玫瑰糠疹 + 史蒂文斯-约翰逊综合征 + Stevens-Johnson综合征 + 中毒性表皮坏死松解症 + SJS + TEN + 伴有嗜酸性粒细胞增多和全身症状的药物反应 + 药物过敏综合征 + DRESS + 急性全身性发疹性脓疱病 + AGEP + 皮肤毛细血管增生症 + RCCEP)) AND (R=(危险因素 + 影响因素 + 高危因素 + 发病因素)) | 222 |
| SinoMed | ("免疫治疗"[标题:智能] OR "免疫检查点抑制剂"[标题:智能] OR "PD-1"[标题:智能] OR "PD-L1"[标题:智能] OR "CTLA-4"[标题:智能] OR "免疫治疗"[摘要:智能] OR "免疫检查点抑制剂"[摘要:智能] OR "PD-1"[摘要:智能] OR "PD-L1"[摘要:智能] OR "CTLA-4"[摘要:智能]) AND ("不良反应"[标题:智能] OR "不良事件"[标题:智能] OR "副作用"[标题:智能] OR "毒副反应"[标题:智能] OR "不良反应"[摘要:智能] OR "不良事件"[摘要:智能] OR "副作用"[摘要:智能] OR "毒副反应"[摘要:智能]) AND ("皮肤反应"[标题:智能] OR "皮肤毒性"[标题:智能] OR "皮肤免疫相关不良事件"[标题:智能] OR "皮疹"[标题:智能] OR "瘙痒"[标题:智能] OR "湿疹"[标题:智能] OR "白癜风"[标题:智能] OR "硬皮病"[标题:智能] OR "牛皮藓"[标题:智能] OR "格罗弗病"[标题:智能] OR "斑丘疹"[标题:智能] OR "类天疱疮"[标题:智能] OR "大疱"[标题:智能] OR "苔藓样"[标题:智能] OR "痤疮样"[标题:智能] OR "丘疹脓疱性"[标题:智能] OR "肉芽肿"[标题:智能] OR "麻疹"[标题:智能] OR "斑秃"[标题:智能] OR "脱发"[标题:智能] OR "毛发红糠疹"[标题:智能] OR "硬皮病"[标题:智能] OR "黏膜炎"[标题:智能] OR "玫瑰糠疹"[标题:智能] OR "史蒂文斯-约翰逊综合征"[标题:智能] OR "Stevens-Johnson综合征"[标题:智能] OR "中毒性表皮坏死松解症"[标题:智能] OR "SJS"[标题:智能] OR "TEN"[标题:智能] OR "伴有嗜酸性粒细胞增多和全身症状的药物反应"[标题:智能] OR "药物过敏综合征"[标题:智能] OR "DRESS"[标题:智能] OR "急性全身性发疹性脓疱病"[标题:智能] OR "AGEP"[标题:智能] OR "皮肤毛细血管增生症"[标题:智能] OR "RCCEP"[标题:智能] OR "皮肤反应"[摘要:智能] OR "皮肤毒性"[摘要:智能] OR "皮肤免疫相关不良事件"[摘要:智能] OR "皮疹"[摘要:智能] OR "瘙痒"[摘要:智能] OR "湿疹"[摘要:智能] OR "白癜风"[摘要:智能] OR "硬皮病"[摘要:智能] OR "牛皮藓"[摘要:智能] OR "格罗弗病"[摘要:智能] OR "斑丘疹"[摘要:智能] OR "类天疱疮"[摘要:智能] OR "大疱"[摘要:智能] OR "苔藓样"[摘要:智能] OR "痤疮样"[摘要:智能] OR "丘疹脓疱性"[摘要:智能] OR "肉芽肿"[摘要:智能] OR "麻疹"[摘要:智能] OR "斑秃"[摘要:智能] OR "脱发"[摘要:智能] OR "毛发红糠疹"[摘要:智能] OR "硬皮病"[摘要:智能] OR "黏膜炎"[摘要:智能] OR "玫瑰糠疹"[摘要:智能] OR "史蒂文斯-约翰逊综合征"[摘要:智能] OR "Stevens-Johnson综合征"[摘要:智能] OR "中毒性表皮坏死松解症"[摘要:智能] OR "SJS"[摘要:智能] OR "TEN"[摘要:智能] OR "伴有嗜酸性粒细胞增多和全身症状的药物反应"[摘要:智能] OR "药物过敏综合征"[摘要:智能] OR "DRESS"[摘要:智能] OR "急性全身性发疹性脓疱病"[摘要:智能] OR "AGEP"[摘要:智能] OR "皮肤毛细血管增生症"[摘要:智能] OR "RCCEP"[摘要:智能]) AND ("危险因素"[摘要:智能] OR "影响因素"[摘要:智能] OR "高危因素"[标题:智能] OR "发病因素"[标题:智能] OR "危险因素"[摘要:智能] OR "影响因素"[摘要:智能] OR "高危因素"[摘要:智能] OR "发病因素"[摘要:智能]) | 33 |
