# Supplementary material for: Risk factors for cutaneous immune-related adverse events: a systematic scoping review
Source: Front Immunol. 2026 Apr 16;17:1722781. doi: 10.3389/fimmu.2026.1722781 (PMC13128564; doi:10.3389/fimmu.2026.1722781)
Supplement: Supplementary file 3 [file Table3.docx]

**Table B.1.** Detailed characteristics of included studies (n=33)

| Included studies | Study design | Groups | Cancer type | ICIs treatment | Age, years M ± SD/[IQR]/(range) | Subtypes of cirAEs | Grade | Occurrence time  M ± SD/[IQR]/(range) | Risk factors | Sample size/risk groups | OR/RR (0.95%CI) | The methods to determine OR/RR |
| --- | --- | --- | --- | --- | --- | --- | --- | --- | --- | --- | --- | --- |
| Abed et al., 2022, Australia^1^ | Case control study | Immune-related adverse events (irAEs)/ non-irAEs group | NSCLC | PD-1/L1 | - | - | - | - | HLA or HLA-I/II alleles has no significant impact on cirAEs toxicity **(P>0.05)** | 156/NA | NA | NA |
| Bai et al., 2021, China^2^ | Case control study | irAEs/ non-irAEs group | Advanced pan-cancer | PD-1/L1， PD-1+CTLA-4 | 61 (24-84) | - | G1~2 | 1.37 (0.43–4.87) months | higher AEC (cutoff = 0.205 × 10^9^/L) | 105/20 | 6.14 (1.81-20.83) | logistic regression analyses |
| Baldini et al., 2020, France^3^ | Retrospective cohort study | Old (≥ 70 years)/ Young (< 70 years) group | Melanoma, NSCLC, urothelial carcinoma, renal cell carcinoma | PD-1/L1, CTLA-4 | Old group: 77 (70-93);  Young group: 59 (17-69) | - | - | - | Age ≥ 70 years old | 603/191 | 2.48 (1.74-3.54) | logistic regression analyses |
| Bastacky et al., 2021, USA^4^ | Case control study | irAEs/ non-irAEs group | Melanoma | PD-1 | 68 (20-91) | - | - | 12 weeks | BMI>25 | 190/137 | 1.97 (1.04-3.74) | - |
| Belzer, 2023, USA ^5^ | Retrospective cohort study | Eczema/ psoriasis/ no dermatologic history group | Pan-cancer | PD-1/L1, CTLA-4, PD-1/L1+CTLA-4 | 66 ± 11 | Psoriasiform, eczematous, lichenoid, maculopapular, vitiligo, pruritus, bullous, acneiform, urticarial, granulomatous, grover’s disease, mucositis, SCAR (severe cutaneous adverse reaction), alopecia, mixed morphology, others | G1:13; G2: 13; G3:1 | - | History of eczema;  History of psoriasis | 287/11; 287/18 | History of eczema: 6.14 (1.79-21.04); History of psoriasis: 19.77 (7.06-55.36) | - |
| Bertrand et al., 2015, France^6^ | Meta-analysis | ipilimumab 3 mg/kg/ ipilimumab 10 mg/kg | Pan-cancer | CTLA-4 | - | - | - | 10 (6-12) weeks | High dose of ipilimumab | 1,265/861 | 3.10 (1.59-6.03) | logistic regression analyses |
| Bottlaender et al., 2020, France ^7^ | Case control study | Cutaneous immune-related adverse events (cirAEs)/ non-cirAEs group | Advanced melanoma | PD-1 | 64.6 [50.0-74.7] | Skin eruption, vitiligo, pruritus, xerosis, sarcoidosis, psoriasis, Grover’s disease | G1-2: 34; G3-4: 5 | 15 [IQR (6–27)] weeks | History of inflammatory/auto-immune disease;  atopy;  Higher eosinophil count (> 0.5 G/L);  longer cycle | 189/14;189/29; 189/21 | History of inflammatory/auto-immune disease: 3.23 (1.07-9.72); atopy: 2.88 (1.27-6.53); Higher eosinophil count: 2.72 (1.07-6.92); longer cycle: 23.64 (3.23-49.45) | logistic regression analyses |
| Bui et al., 2022, USA ^8^ | Retrospective cohort study | Female/ male group | Metastatic melanoma | PD-1, PD-1+CTLA-4 | Female group: 60 (22-88);  Male group: 65 (30-91) | Maculopapular, pruritus, vitiligo, non-specific rash, Lichenoid, Acneiform/papulopustular | - | - | Female,  Post-menopausal woman (age ≥52) | 235/93; 235/27 | Female: 2.11 (1.17-3.82)  post-menopausal woman (age≥52): 2.17 (1.00-4.69) | logistic regression |
| Chen et al., 2023, China^9^ | Case control study | irAEs/ non-irAES | NSCLC | PD-1 | - | Maculopapular, pruritus | G1-2: 63; G3:3 | - | Smoking history, 2 cycles or over treatments, Combined with targeted therapy | 261/130; 261/231; 261/22 | Smoking history: 2.75 (1.59-4.75); 2 cycles or over treatment: 6.84 (2.47-18.95); Combined with targeted therapy: 34.11 (4.21-276.18) | logistic regression |
| Cortellini et al., 2020, Italy ^10^ | Retrospective cohort study | Underweight (BMI ≤ 18.5)/normal weight (BMI 18.5-25)/overweight (BMI 25-30)/obsess(BMI ≥ 30) | Measurable stage IV solid cancer | PD-1/L1 | 68 (21-92) | - | - | - | BMI > 25 | 1,070/416; 1,070/130 | Overweight (25<BMI≤29.9): 24.7 (20.2-30.0)  Obese (BMI > 30): 17.7 (11.2-26.5) | Logistic regression |
| Darwin et al., 2022, USA ^11^ | Retrospective cohort study | Adolescents and young adults(AYA, 15-39)/elderly patients (> 65) group | melanoma patients | PD-1, PD-1+CTLA-4 | AYA cohort: 31.2 (18.7–39.9);  Elderly cohort: 72.0 (65.1–89.1) | Maculopapular, Vitiligo or psoriasis | G1~G2 | - | Elderly patients ≥ 65 (Maculopapular) | 153/105 | 2.77 (1.36-5.66) | logistic regression analyses |
| Dousset et al., 2021, France ^12^ | Case control study | Vitiligo/ non-vitiligo group | Melanoma | PD-1 | 66 (24-94) | Vitiligo | - | 6.9 months (range, 1-35.5 months) | Male | 457/262 | 1.66 (1.00-2.78) | logistic regression analyses |
| Duma et al., 2019, USA^13^ | Case control study | Male/ female | Melanoma, NSCLC | PD-1 | - | - | - | - | Male | 476/259 | 2.30 (1.25-4.22) | - |
| Fujimoto et al., 2021, Japan^14^ | Case control study | NLR < 2.86/ NLR ≥ 2.86 | NSCLC | PD-1/L1 | 68 (45–87) | - | 1-2 | - | NLR < 2.86 | 115/45 | 2.69 (1.21-6.01) | logistic regression |
| Fujimura et al., 2020, Japan ^15^ | Retrospective cohort study | Vitiligo/ non-vitiligo group | Melanoma | PD-1 | - | Vitiligo | - | - | Growth value from baseline to day 42 of CCL19 (threshold = 132.9pgmL^-1^) | 57/19 | P < 0.001 | - |
| Ge et al., 2021, China^16^ | Meta-analysis | - | Pan-cancer | PD-1/L1, CTLA-4, PD-1/L1+CTLA-4 | - | Maculopapular, pruritus | G1-2:6,966; G3-4:342 | - | PD-1/-L1+CTLA-4 than monotherapy;  CTLA-4 than PD-1/L1 | 29,941/12,044; 29,941/1,085 | PD-1/L1+CTLA-4: 2.27 (1.70-3.02) (maculopapular);  1.60 (1.27-2.01) (pruritus)  CTLA-4: 1.83 (1.51-2.21) (pruritus) | the fixed-effect model |
| Hasan Ali et al., 2019, Switzerland^17^ | Case control study | IrAEs group/ non-irAEs group | Metastatic NSCLC, metastatic melanoma | PD-1/L1, CTLA-4, PD-1/L1+CTLA-4 | 68 (62-74) | Pruritus, Maculopapular | - | - | Pruritus: HLADRB1*11:01 | 102/95 | OR = 4.53, X^2^_1 , 95_= 9.45, P = 0.002 |  |
| Hasan Ali et al., 2020, Switzerland ^18^ | Case control study | CirAEs group/ non-cirAEs group | NSCLC | PD-1/L1 | 67 | Maculopapular, pruritus | 1~2 | - | high anti-BP180 IgG | 40/17 | 4.05 (1.12-14.66) | - |
| He et al., 2023, China^19^ | Case control study | RCCEP/ non-RCCEP | NSCLC | PD-1 | - | RCCEP | G1-2: 20; G3-4: 4 | 10.5 w | eosinophil (EOS%) >1.75% | 80/34 | 4.48 (1.14-17.65) | logistic regression |
| Jacoby et al., 2022, USA^20^ | Case control study | CirAEs/ non-cirAEs group | Pan-cancer | PD-1/L1, CTLA-4, PD-1/L1+CTLA-4 | CirAEs group: 65 [55-73];  Non-cirAEs group: 67 [58-74] | Rash (not otherwise specified), maculopapular, pruritus, bullous, SCAR, vitiligo | G1-2: 18; G3-4: 5 | - | cirAEs: a history of three or more drug allergies; history of penicillin allergy  Nonspecific rash: at least one drug allergy  Pruritus: history of anaphylaxis resulting from medications | 2,459/23; 2,459/53; 2,459/227; 2,459/30 | a history of three or more drug allergies: 6.57 (1.77-24.42) (cirAEs); history of penicillin allergy: 3.12 (1.6-8.35) (cirAEs); at least one drug allergy: 1.95 (1.12-3.38) (nonspecific rash); history of anaphylaxis: 6.81 (1.33-34.67) (pruritus) | logistic regression |
| Kobayashi et al., 2020, Japan ^21^ | Case control study | CirAEs/ non-cirAEs group | RCC | PD-1 | 67 (41-85) | - | 1-2 | 23 (15-288) days | PLR<156 | 53/17 | 6.15 (1.67-22.56) | logistic regression |
| Leung et al., 2023, USA^22^ | Retrospective cohort study | ICI + TVEC/ ICI group | Cutaneous malignancy | PD-1/L1, CTLA-4, PD-1/L1+CTLA-4 | ICI + TVEC: 68.2;  ICI: 65.1 | - | - | ICI: 55(21,160) days;  ICI+TVEC: 124 (38,373) days | TVEC | 892/93 | 2.03 (1.22-3.36) | the time-varying Cox proportional hazard model |
| Lewinson et al., 2021, Canada^23^ | Case control study | CirAEs/ non-cirAEs group | Metastatic melanoma, NSCLC | PD-1 | CirAEs group: 68.1 ± 11.5;  Non-cirAEs group: 64.8 ± 11.3 | - | - | - | Higher age;  Baseline lower NLR;  Baseline lower lactate dehydrogenase  ECOG status 0-1 | ECOG status 0-1: 410/286 | p = 0.0467; p = 0.013; p = 0.008; ECOG status 0-1:3.29 (1.45-7.44) | - |
| Li, 2021, China ^24^ | Case control study | CirAEs/ non-cirAEs group | Pan-cancer | PD-1 | 61 (32-85) | Maculopapular, pruritus, RCCEP, psoriasis | G1-2: 58; G3-4: 4 | 81(9-653) days | Previous chemotherapy, target treatment, combined chemotherapy, target treatment no significant different |  | NA | NA |
| Luangnara et al., 2022, Italy ^25^ | ambispective cohort study | CirAE/ non-cirAE group | Pan-cancer | PD-1/L1, CTLA-4, PD-1/L1+CTLA-4 | 65.9 ± 10.3 | - | 1-2 | 67 (21-146) days | Age > 75 years old  History of chronic kidney disease stage 3-4 | 112/16; 112/4 | Age > 75: 2.13 (1.09-4.15); History of chronic kidney disease stage 3-4: 3.52 (2.33-5.31) | Poisson regression models |
| Lv et al., 2021, China ^26^ | Case control study | CirAE/ non-cirAE group | NSCLC | PD-1 | - | Maculopapular, pruritus, mucositis | G1-2: 4; G3-4: 3 | - | smoking | 42/18 | 1.87 (1.02-3.43) | logistic regression |
| Ma et al., 2022, China ^27^ | Case control study | irAE/ non-irAE group | Pan-cancer | PD-1/L1 | 62 (30-80) | Maculopapular, pruritus, psoriasis, RCCEP | 1-2 | - | ECOG PS 0-1 | 95/86 | P= 0.046 | - |
| Madjar et al., 2023, Switzerland ^28^ | Meta analysis | - | Pan-cancer | PD-L1 | - | - | 1-2 | - | Asian ancestry, high BMI | 10,344/1,605; 10,344/- | Asian ancestry: 1.82 (1.62-2.04); high BMI: 1.09 (1.04-1.13) | Cox proportional hazards regression model |
| Nguyen et al., 2023, USA ^29^ | Retrospective cohort study | Non-acral cutaneous melanoma/ acral melanoma/ mucosal melanoma/ uveal melanoma/ melanoma of unknown primary group | Metastatic melanoma | PD-1/L1, CTLA-4, PD-1/L1+CTLA-4 | Non-acral cutaneous melanoma: 64.8 [56.2, 73.9]  Acral melanoma: 67.2 [56.1, 76.7];  Mucosal melanoma: 65.4 [59.0, 74.4]  Uveal melanoma: 65.9 [58.5–69.8]  Melanoma of unknown primary: 63.8 [54.0–72.3] | Rash NOS, Drug Hypersensitivity, Eczematous Eruption, Lichenoid Eruption, Maculopapular Eruption, Isolated Pruritus, Vitiligo, Other (acneiform eruption, bullous eruption, erythemamultiforme-like eruption, mucositis, panniculitis, psoriasiform eruption, and SJS/TEN-like eruption.) | - | 0.1 (0.0-0.5) year | Non-acral cutaneous melanoma | 747/478 | 2.44 (1.18-5.00) | Weibull accelerated failure time regression model |
| Nikolaou et al., 2022, Italy ^30^ | Retrospective cohort study | Ezema/ psoriasis/ eczema/ pruritus/ lichenoid/ vitiligo/ uncommon toxicities group | Pan-cancer | PD-1/L1, CTLA-4, PD-1/L1+CTLA-4 | Men: 66.3±11.5;  Women: 61.8±12.4 | Eczematous reaction, Maculopapular, psoriasis, lichen planus-like rash, Pruritus, Vitiligo, Bullous pemphigoid, Uncommon | G1:453; G2:370; G3: 140; G4: 4 | 20.1 (3~138) weeks | Pruritus (compared with psoriasis, lichenoid and eczematous reactions): ICIs + chemotherapy (compared with ICIs);  macular rash, vitiligo or multiple toxicities (compared with pruritus)：melanoma (compared with NSCLC);  pruritus (compared with macular rash and vitiligo): CTLA-4 (compared with PD-1) | 762/57; 762/199; 762/20 | ICIs + chemotherapy (compared with ICIs): 12.5 (3.23-50) (pruritus vs psoriasis); 6.67 (1.30-3.33) (pruritus vs lichenoid); 4.17 (1.28-14.29) (pruritus vs eczematous reactions); melanoma (compared with NSCLC): 3.63 (1.23-10.7) (macular rash vs pruritus); 91 (9.80-852) (vitiligo vs pruritus); 3.73 (1.26-11.00) (multiple toxicities vs pruritus); CTLA-4 (compared with PD-1): 9.09 (1.32-100) (pruritus vs macular rash and vitiligo); 14.29 (1.28-166.67) (pruritus vs vitiligo) | logistic regression analyses |
| Paderi et al., 2021, Italy ^31^ | Retrospective cohort study | Elderly (≥ 70 years/ younger group | NSCLC, melanoma and RCC | PD-1/L1 | 67.5 (27-91) | - | - | - | Elderly (≥ 70 years) | 146/89 | 4.22 (1.57-11.31) | logistic regression analyses |
| Pan et al., 2023, USA ^32^ | Retrospective cohort study | Pembrolizumab/ nivolumab/ ipilimumab/ ipi-nivo group | melanoma | PD-1, CTLA-4 | 63.8 | - | - | - | The treatment regimen has no significant impact on cirAEs toxicity (P>0.05) | NA | NA | NA |
| Said et al., 2022, USA ^33^ | Cohort study | BP/ non-BP group | Pan-cancer | PD-1/L1, CTLA-4, PD-1/L1+CTLA-4 | BP group: 72.8 [13.4];  Non-BP group: 65.5 [15.72] | BP | - | - | BP：Age ≥ 70, melanoma, nonmelanoma skin cancer | 2,955/1,063; 2,955/505; 2,955/75 | 2.32 (1.19-4.59); 3.21 (1.15-6.58); 8.32 (2.81-21.13) | logistic regression analyses |
| Stephens et al., 2023, USA^34^ | Cohort study | CirAEs/ non-cirAEs group | melanoma | PD-1/L1, CTLA-4, PD-1/L1+CTLA-4 | CirAE group: 66 [56-75];  Non-cirAE group: 63 [56-75] | Maculopapular or papulopustular, Eczematous, lichenoid, isolated pruritus, psoriasiform, vitiligo, other (erythema multiforme, bullous eruption, DRESS, macular erythema, morbilliform, urticarial) | 1-2 | 42 (16-119) days | Tumor-infiltrating lymphocytes (TIL) status at baseline had no significant with cirAEs, or its subtypes. | NA | NA | NA |
| Storm et al., 2022, Netherlands ^35^ | Case control study | ≥ 65/ <65 year | Pan-cancer | PD-1/L1 | 66.0 (28-86) | - | 1-2 | - | ≥ 65 year | 217/125 | 1.79 (1.02-3.12) | logistic regression analyses |
| Tang et al., 2020, China ^36^ | Cohort study | Rash/ non-rash group;  Pruritus/ non-pruritus group | NSCLC | PD-1 | - | Maculopapular, pruritus | G1-2:54; G3: 1 | - | Maculopapular: female, allergy history; TNM stage II or above, chemotherapy history;  Pruritus: allergy history | 110/24; 110/12; 110/81; 110/28 | Female: 4.33 (1.68-11.19) (Maculopapular);  allergy history: 80.3 (4.58-1409) (Maculopapular), 266.2 (14.5-4891) (Pruritus)  TNM stage II or above: 4.50 (1.44-14.02) (Maculopapular)  chemotherapy history: 2.91 (1.19-7.08) (Maculopapular) | - |
| Thompson et al., 2021, USA ^37^ | Retrospective cohort study | Adult (> 18)/ pedoatric group | sarcomas | PD-1/L1, CTLA-4, PD-1/L1+CTLA-4 | Adult: 55[28-66];  Pediatric: 16 [11-18] | Acneiform reaction, maculopapular reaction, isolated xerosis, pruritus without visible rash, psoriasiform eruption | 1-2 | 35 (28–84) days | No significant differences between children and adults of cirAEs (P>0.05) |  | NA | NA |
| Thompson et al., 2021, USA^38^ | Retrospective cohort study | White/ nonwhite group | Pan-cancer | PD-1/PD-L1 | 65 [57-73] | NOS, pruritus, eczematous, lichenoid, psoriasiform, other (vitiligo, erythema multiforme, bullous, mucositis, panniculitis, SJS/TEN, Sweet syndrome) | 1-2 | 51 (19-141) days | White patients | 2,447/2,189 | 2.00 (1.10-3.60) | logistic regression analyse |
| Tyan et al., 2021, USA ^39^ | Case control study | cirAEs/ non-cirAEs | melanoma | PD-1/L1, CTLA-4, PD-1/L1+CTLA-4 | 70.5 [29–91] | - | 1-2 | 7.08 (0.1-27.7) months | Baseline high Ang-1 and CD40L | 52/- | p = 0.005; p = 0.006 | - |
| Wan et al., 2023, USA^40^ | Cohort study | With pre-existing inflammatory diseases/ without pre-existing inflammatory diseases group | Pan-cancer | PD-1/L1, CTLA-4, PD-1/L1+CTLA-4 | 64.2 ± 13.0 | - | - | 64 [21, 175] days | Pre-existing cutaneous inflammatory diseases;  Pre-existing atopic dermatitis;  Pre-existing morphea;  Pre-existing psoriasis;  Pre-existing pemphigus | 3,607/1,354; 3,607/62; 3,607/20 | Pre-existing cutaneous inflammatory diseases: 1.56 (1.25-1.94);  Pre-existing atopic dermatitis : 3.58 (2.34-5.48);  Pre-existing morphea: 3.23 (1.42-7.37);  Pre-existing psoriasis: 1.79 (1.23-2.62);  Pre-existing pemphigus: 35.2 (4.08-304) | Cox Proportional Hazard (CoxPH) models |
| Wang et al., 2022, China^41^ | Case control study | IrAEs/ non-irAEs | Gastrointestinal cancer | PD-1/L1 | 52 (22-77) | - | - | - | the higher baseline levels of BTLA, GM-CSF, IL-4, PD-1, PD-L1, and TIM-3 | - | P = 0.027, P = 0.031, P = 0.01, P =0.019, P = 0.022, P = 0.043 | - |
| Wongvibulsin et al., 2022, USA^42^ | Retrospective cohort study | CirAEs/ non-cirAEs group | Pan-cancer | PD-1/L1, CTLA-4, PD-1/L1+CTLA-4 | 67.5 ± 11.8 | Rash and other nonspecific eruption, pruritus, drug eruption or other nonspecific drug reaction, mucositis, erythroderma, maculopapular eruption, vitiligo, lichen planus, BP, Grover’s disease | G1-2: 1062; G3-4:109 | 113 (42-254) days | Melanoma and renal cell carcinoma (compared with lung cancer);  Pembrolizumab therapy compared with ipilimumab therapy;  Combination therapy (anti CTLA-4 and anti PD-1/PD-L1) compared with pembrolizumab | 8,637/2,299;  8,637/1,059  8,637/- | Melanoma (vs lung cancer): 2.47 (2.11-2.89); renal cell carcinoma (vs lung cancer): 1.65 (1.36-2.00)  Pembrolizumab therapy (vs ipilimumab): 1.28 (1.02-1.61);  Combination therapy (anti CTLA-4 and anti PD-1/PD-L1) compared with pembrolizumab: 1.65 (1.36-2.00) | logistic regression |
| Xu et al., 2023, China ^43^ | Case control study | irAEs group/ non-irAEs group | NSCLC | PD-1/L1 | - | - | 1-2 | 63d | higher pretreatment absolute lymphocyte count (ALC) | 130/83 | 220 (1.34-7.61) | logistic regression |
| Xu et al., 2023, China^44^ | Retrospective cohort study | CirAEs group/ non-cirAEs group | Gastric and esophageal cancer | PD-1 | 66 (27-89) | - | - | - | PD-L1 expression ≥ 1%;  No corticosteroid used;  Baseline CD4+/CD8+ ratio < 1.10 | 151/52;  151/98;  151/- | PD-L1 expression ≥ 1%:  6.21 (2.37-16.27);  No corticosteroid used: 3.17 (1.10-9.17);  Baseline CD4+/CD8+ ratio < 1.10: 3.27 (1.43-7.46) | logistic regression |
| Xu et al., 2022, China ^45^ | Case control study | Rash/ non-rash group;  Pruritus/ non-pruritus group | NSCLC | PD-1 | - | Maculopapular, pruritus | G1-2:14; G3:1 | - | Maculopapular: smoking history; smoking index > 400 | 110/93; 110/100 | 100 (9.09-1000); 4.35 (1.11-16.67) | logistic regression |
| Yang et al., 2023, China^46^ | Retrospective cohort study | With aspirin/ without aspirin | Pan-cancer | PD-1/L1, CTLA-4, PD-1/L1+CTLA-4 | With aspirin: 68.9 [9.0];  Without aspirin: 63.9 [12.5] | Pruritus, psoriasis, Maculopapular, SJS, vitiligo | - | - | Maculopapular, SJS, vitiligo: not use aspirin | 123,104/5,359 | 1.47 (1.22-1.79); 5.56 (1.79-16.67); 2.86 (1.05-7.69) | logistic regression |
| Yu et al., 2023, China ^47^ | Case control study | CirAEs / non-cirAEs group | Pan-cancer | PD-1 | 62.16±10.35 | RCCEP, maculopapular, pruritus, Blister | G1-2: 76; G3-4: 7 | - | Not combined with antiangiogenic drugs | 528/232 | 1.13 (1.09-1.17) | logistic regression |
| Zamora et al., 2021, Spain ^48^ | Case control study | CD4 + PLT + high percentage group / CD4 + PLT + low percentage group | NSCLC | PD-1/L1 | 66.29 [36.98–85.34] | Maculopapular, pruritus, Vitiligo | 1-2 | 6.22 (0.28-145) weeks | CD4 + PLT + high percentage of circulating leukocyte–PLT complexes | 87/20 | 5.67 (1.49-21.54) | - |
| Zhang et al., 2022, China^49^ | retrospective cohort study |  | esophageal, gastric, or colon cancer | PD-1/L1, CTLA-4, PD-1/L1+CTLA-4 | 58 (18-85) | Maculopapular, pruritus | G1-2: 48; G3-4: 4 | - | ECOG PS 0-1, low NLR | 243/237;  243/- | 1.75 (1.02-2.99);  1.12 (1.00-1.25) | - |
| Zhao et al., 2023, China^50^ | Retrospective cohort study | CirAEs / non-cirAEs group | Pan-cancer | PD-1 | 63.5 ± 9.8 | RCCEP, pruritus, maculopapular, vitiligo, TEN, psoriasis | G1-2: 100; G3-4: 1 | - | RCCEP: camrelizumab | 456/149 | 8.40 (5.15-13.72) | logistic regression |

ICI(s), immune checkpoint inhibitor(s). PD-(L)-1, programmed cell death (ligand)-1. CTLA-4, cytotoxic T lymphocyte antigen-4. irAE(s): immune-related adverse event(s). CirAEs, cutaneous immune-related adverse events. SJS/TEN, Stevens-Johnson syndrome/toxic epidermal necrolysis. RCCEP, reactive cutaneous capillary endothelial proliferation. DRESS, drug reaction with eosinophilia and systemic symptoms. IQR, interquartile range. G, grade. CTCAE, Common Terminology Criteria for Adverse Events. V: version. AEC, absolute eosinophil count. BP, bullous pemphigoid. TVEC, talimogene laherparepvec. NLR, neutrophil to lymphocyte ratio. ECOG, Eastern Cooperative Oncology Group. OR, odds ratio. BTLA, B- and T-lymphocyte attenuator. GM-CSF, granulocyte-macrophage colony stimulating factor. IL-4, interleukin-4. TIM-3, T cell immunoglobulin and mucin domain 3. -, not-reported. NA, not applicable.

1. Abed A, Law N, Calapre L, et al. Human leucocyte antigen genotype association with the development of immune-related adverse events in patients with non-small cell lung cancer treated with single agent immunotherapy. *European Journal of Cancer.* 2022;172:98-106.

2. Bai Rilan, Chen Naifei, Chen Xiao, et al. Analysis of characteristics and predictive factors of immune checkpoint inhibitor-related adverse events. *Cancer Biol Med.* 2021;18(4):1118-1133.

3. Baldini C, Martin Romano P, Voisin A-L, et al. Impact of aging on immune-related adverse events generated by anti-programmed death (ligand)PD-(L)1 therapies. *European journal of cancer (Oxford, England : 1990).* 2020;129:71-79.

4. Bastacky ML, Wang H, Fortman D, et al. Immune-Related Adverse Events in PD-1 Treated Melanoma and Impact Upon Anti-Tumor Efficacy: A Real World Analysis. *Frontiers in oncology.* 2021;11.

5. Belzer A. *Characterization of Cutaneous Immune-Related Adverse Events Due to Immune Checkpoint Inhibitors: A Retrospective Analysis Performed at the Smilow Cancer Center Oncodermatology Clinic* [M.D.]. United States -- Connecticut, Yale University; 2023.

6. Bertrand A, Kostine M, Barnetche T, Truchetet ME, Schaeverbeke T. Immune related adverse events associated with anti-CTLA-4 antibodies: systematic review and meta-analysis. *BMC medicine.* 2015;13:211.

7. Bottlaender L, Amini-Adle M, Maucort-Boulch D, Robinson P, Thomas L, Dalle S. Cutaneous adverse events: a predictor of tumour response under anti-PD-1 therapy for metastatic melanoma, a cohort analysis of 189 patients. *Journal of the European Academy of Dermatology and Venereology.* 2020;34(9):2096-2105.

8. Bui AN, Bougrine A, Buchbinder EI, Giobbie-Hurder A, LeBoeuf NR. Female sex is associated with higher rates of dermatologic adverse events among patients with melanoma receiving immune checkpoint inhibitor therapy: A retrospective cohort study. *Journal of the American Academy of Dermatology.* 2022;87(2):403-406.

9. Chen X M, Chen S J, Gan B, Qiu J, Cui H, H YH. Analysis of the Status and Influencing Factors of Immune⁃Related Adverse Events in Advanced Non⁃Small⁃Cell Lung Cancer Patients. *J Evid-Based Med.* 2023;23(03):174-180.

10. Cortellini A, Bersanelli M, Santini D, et al. Another side of the association between body mass index (BMI) and clinical outcomes of cancer patients receiving programmed cell death protein-1 (PD-1)/ Programmed cell death-ligand 1 (PD-L1) checkpoint inhibitors: A multicentre analysis of immune-related adverse events. *European journal of cancer (Oxford, England : 1990).* 2020;128:17-26.

11. Darwin A, Skinner A, Reed DR, Tanvetyanon T. Immune-Related Toxicity Among Adolescent and Young Adult with Melanoma as Compared with the Elderly. *Journal of adolescent and young adult oncology.* 2022;11(6):550-555.

12. Dousset L, Pacaud A, Barnetche T, et al. Analysis of tumor response and clinical factors associated with vitiligo in patients receiving anti-programmed cell death-1 therapies for melanoma: Across-sectional study. *JAAD international.* 2021;5:112-120.

13. Duma N, Abdel‐Ghani A, Yadav S, et al. Sex Differences in Tolerability to Anti‐Programmed Cell Death Protein 1 Therapy in Patients with Metastatic Melanoma and Non‐Small Cell Lung Cancer: Are We All Equal? *The oncologist.* 2019;24(11):e1148-e1155.

14. Fujimoto A, Toyokawa G, Koutake Y, et al. Association between pretreatment neutrophil-to-lymphocyte ratio and immune-related adverse events due to immune checkpoint inhibitors in patients with non-small cell lung cancer. *Thoracic cancer.* 2021;12(15):2198-2204.

15. Fujimura T, Tanita K, Sato Y, et al. Immune checkpoint inhibitor-induced vitiligo in advanced melanoma could be related to increased levels of CCL19. *The British journal of dermatology.* 2020;182(5):1297-1300.

16. Ge Y, Zhang H, Weygant N, Yao J. Differential Dermatologic Adverse Events Associated With Checkpoint Inhibitor Monotherapy and Combination Therapy: A Meta-Analysis of Randomized Control Trials. *Frontiers in Pharmacology.* 2021;12.

17. Hasan Ali O, Berner F, Bomze D, et al. Human leukocyte antigen variation is associated with adverse events of checkpoint inhibitors. *European journal of cancer (Oxford, England : 1990).* 2019;107:8-14.

18. Hasan Ali O, Bomze D, Ring SS, et al. BP180-specific IgG is associated with skin adverse events, therapy response, and overall survival in non-small cell lung cancer patients treated with checkpoint inhibitors. *Journal of the American Academy of Dermatology.* 2020;82(4):854-861.

19. He X, Fang J, Yu P, et al. Risk of reactive cutaneous capillary endothelial proliferation induced by camrelizumab in patients with non-small cell lung cancer: a retrospective study. *Journal of Thoracic Disease.* 2023;15(12):6687-6696.

20. Jacoby TV, Otto TS, Asdourian MS, et al. Association of pre-existing drug allergies with cutaneous immune-related adverse events among patients on immune checkpoint inhibitor therapy. *Br J Dermatol.* 2022;187(3):424-426.

21. Kobayashi K, Iikura Y, Hiraide M, et al. Association between immune-related adverse events and clinical outcome following nivolumab treatment in patients with metastatic renal cell carcinoma. *In vivo (Athens, Greece).* 2020;34(5):2647-2652.

22. Leung BW, Wan G, Nguyen N, et al. Increased risk of cutaneous immune- related adverse events in patients treated with talimogene laherparepvec and immune checkpoint inhibitors: A multi-hospital cohort study. *Journal of the American Academy of Dermatology.* 2023;88(6):1265-1270.

23. Lewinson RT, Meyers DE, Vallerand IA, et al. Machine learning for prediction of cutaneous adverse events in patients receiving antiePD-1 immunotherapy. *JOURNAL OF THE AMERICAN ACADEMY OF DERMATOLOGY.* 2021;84(1):183-185.

24. Li L. *Clinical study on the side-effects of PD-1 inhibitors in the treatment of advanced cancers* [M.D.], Sichuan University; 2021.

25. Luangnara A, Kiratikanon S, Ketpueak T, et al. Incidence and factors associated with cutaneous immune-related adverse events to immune check point inhibitors: An ambispective cohort study. *Frontiers in immunology.* 2022;13:965550.

26. Lv W Y, S ZS. Analysis of Factors Influencing Drug-Related Adverse Reactions in PD-1 Immunotherapy for Non-Small Cell Lung Cancer. *China Pract Med.* 2021;16(31):134-136.

27. Ma Y, Ma X, Wang J, Wu S, Wang J, Cao B. Absolute eosinophil count may be an optimal peripheral blood marker to identify the risk of immune-related adverse events in advanced malignant tumors treated with PD-1/PD-L1 inhibitors: a retrospective analysis. *World Journal of Surgical Oncology.* 2022;20(1).

28. Madjar K, Mohindra R, Durán-Pacheco G, et al. Baseline risk factors associated with immune related adverse events and atezolizumab. *Frontiers in oncology.* 2023;13:1138305.

29. Nguyen N, Wan G, Ugwu-Dike P, et al. Influence of melanoma type on incidence and downstream implications of cutaneous immune-related adverse events in the setting of immune checkpoint inhibitor therapy. *Journal of the American Academy of Dermatology.* 2023;88(6):1308-1316.

30. Nikolaou VA, Apalla Z, Carrera C, et al. Clinical associations and classification of immune checkpoint inhibitor‐induced cutaneous toxicities: a multicentre study from the European Academy of Dermatology and Venereology Task Force of Dermatology for Cancer Patients. *British Journal of Dermatology.* 2022;187(6):962-969.

31. Paderi A, Fancelli S, Caliman E, et al. Safety of immune checkpoint inhibitors in elderly patients: An observational study. *Current Oncology.* 2021;28(5):3259-3267.

32. Pan CX, Kim DY, Lau CB, et al. Comparative analysis of immune-related adverse events among patients with melanoma on immune checkpoint inhibitors: a retrospective cohort study. *The British journal of dermatology.* 2023;189(5):637-640.

33. Said JT, Liu M, Talia J, et al. Risk Factors for the Development of Bullous Pemphigoid in US Patients Receiving Immune Checkpoint Inhibitors. *JAMA dermatology.* 2022;158(5):552-557.

34. Stephens MR, Asdourian MS, Jacoby TV, et al. Tumor-infiltrating lymphocytes as a predictive biomarker of cutaneous immune-related adverse events after immune checkpoint blockade in patients with advanced melanoma. *Journal of the American Academy of Dermatology.* 2023;89(1):140-142.

35. Storm BN, Abedian Kalkhoran H, Wilms EB, et al. Real-life safety of PD-1 and PD-L1 inhibitors in older patients with cancer: An observational study. *Journal of geriatric oncology.* 2022;13(7):997-1002.

36. Tang S, Hou L, Wang H. Longitudinal study of skin toxicity caused by immunotherapy in patients with non-small lung cancer. *J Nurs Sci.* 2020;35(16):35-37.

37. Thompson LL, Chang MS, McCormack L, et al. Patterns of cutaneous immune-related adverse events in adults and children with advanced sarcoma: a retrospective cohort study. *The British journal of dermatology.* 2021;184(2):363-365.

38. Thompson LL, Pan CX, Chang MS, Krasnow NA, Blum AE, Chen ST. Impact of ethnicity on the diagnosis and management of cutaneous toxicities from immune checkpoint inhibitors. *Journal of the American Academy of Dermatology.* 2021;84(3):851-854.

39. Tyan K, Baginska J, Brainard M, et al. Cytokine changes during immune-related adverse events and corticosteroid treatment in melanoma patients receiving immune checkpoint inhibitors. *Cancer Immunology, Immunotherapy.* 2021;70(8):2209-2221.

40. Wan G, Nguyen N, Leung BW, et al. Pre-Existing Inflammatory Disease Predicts Cutaneous Immunotherapy Toxicity Development: A Multi-Institutional Cohort Study. *MedRxiv : the preprint server for health sciences.* 2023.

41. Wang Y, Zou J, Li Y, et al. Serological biomarkers predict immune-related adverse events and clinical benefit in patients with advanced gastrointestinal cancers. *Frontiers in immunology.* 2022;13:987568.

42. Wongvibulsin S, Pahalyants V, Kalinich M, et al. Epidemiology and risk factors for the development of cutaneous toxicities in patients treated with immune-checkpoint inhibitors: A United States population-level analysis. *Journal of the American Academy of Dermatology.* 2022;86(3):563-572.

43. Xu J H, Xu G J, Duan L F, et al. The risk factor analysis for immune-related adverse events in non-small cell lung cancer patients treated with immune checkpoint inhibitors and their relationship to effectiveness. *Tianjin Med J.* 2023;51(02):207-212.

44. Xu S, Zhu Q, Wu L, et al. Association of the CD4+/CD8+ ratio with response to PD-1 inhibitor-based combination therapy and dermatological toxicities in patients with advanced gastric and esophageal cancer. *International Immunopharmacology.* 2023;123:N.PAG-N.PAG.

45. Xu X, Tang s, Zhang m, Guan s. Influencing factors of common adverse reactions of immunotherapy in patients with non-small cell lung cancer. *J Bengbu Med Coll.* 2022;47(08):1096-1101.

46. Yang H, Liu Z, Li R, Huang R, Peng X. The association between aspirin use and immune-related adverse events in specific cancer patients receiving ICIs therapy: analysis of the FAERS database. *Frontiers in pharmacology.* 2023;14:1259628.

47. Yu M, X L. Immunerelated adverse events and risk factors of carrelizumab in 528 cases of cancer patients. *Chinese Journal of Pharmacovigilance.* 2023;20(10):1134-1140.

48. Zamora C, Riudavets M, Anguera G, et al. Circulating leukocyte–platelet complexes as a predictive biomarker for the development of immune-related adverse events in advanced non-small cell lung cancer patients receiving anti-PD-(L)1 blocking agents. *Cancer Immunology, Immunotherapy.* 2021;70(6):1691-1704.

49. Zhang Z, Xie T, Qi C, Zhang X, Shen L, Peng Z. Peripheral Blood Biomarkers Predictive of Efficacy Outcome and Immune-Related Adverse Events in Advanced Gastrointestinal Cancers Treated with Checkpoint Inhibitors. *Cancers.* 2022;14(15):3736.

50. Zhao Y, Guan F, Han Z, Zhang X. Clinical observation on skin adverse reactions after treatment of programmed cell death protein-1 inhibitors. *J Army Med Univ.* 2023;45(22):2352-2357.
